# The expression and significance of AKR1B10 in laryngeal squamous cell carcinoma

**DOI:** 10.1038/s41598-021-97648-y

**Published:** 2021-09-14

**Authors:** Jixuan Liu, Hongyan Ban, Yafang Liu, Jinsong Ni

**Affiliations:** 1 Department of Pathology, The First Hospital of Jilin University, Changchun, 130021 Jilin China; 2 Department of Pathology, Maternal and Child Health Hospital of Jilin Province, Changchun, 130021 Jilin China; 3 The Key Laboratory of Pathobiology, Ministry of Education, Jilin University, Changchun, 130021 China

**Keywords:** Cancer, Cell biology

## Abstract

Aldosterone reductase family 1 member B10 (AKR1B10) is a nicotinamide adenine dinucleotide phosphate (reduced coenzyme II)-dependent oxidoreductase, and its biological functions include carbonyl detoxification, hormone metabolism, osmotic adjustment, and lipid synthesis. Studies suggested that AKR1B10 is a new biomarker for cancer based on its overexpression in epithelial tumors, such as breast cancer, cervical cancer, and lung cancer. At present, studies on the expression of AKR1B10 in laryngeal cancer have not been reported. However, we found that AKR1B10 is upregulated in laryngeal carcinoma, and its expression was negatively correlated with the degree of differentiation. In addition, AKR1B10 expression was positively correlated with tumor size; lymph node metastasis; alcohol use; and Ki-67, mutant p53, and matrix metalloproteinase 2 expression. AKR1B10 was overexpressed in Hep-2 laryngeal carcinoma cells. Oleanolic acid inhibited AKR1B10 activity and expression in Hep-2 cells and suppressed Hep-2 cell proliferation, migration, and invasion. Therefore, AKR1B10 may be related to the development of laryngeal carcinoma, suggesting its use as a prognostic indicator for laryngeal cancer.

## Introduction

Laryngeal squamous cell carcinoma (LSCC) is a malignant tumor originating from the epithelial tissue of the laryngeal mucosa. Although significant progress has been made in the development of surgery and radiotherapy in recent decades, the mortality rate of LSCC remains high^1^. Therefore, exploring the molecular mechanism of the occurrence and development of LSCC and developing new treatment options are current research hotspots. Aldosterone reductase family 1 member B10 (AKR1B10), which belongs to the AKR1B subfamily, is also known as aldose reductase-like enzyme, and it is present in limited tissues in humans, primarily the small intestine and colon. AKR1B10 is also expressed in the liver, thymus, prostate, and testis. Conversely, AKR1B10 is absent in other tissues (e.g., brain, spleen, heart, ovaries, lungs, placenta, pancreas, skeletal muscle, kidneys)^2^. AKR1B10 protein is a nicotinamide adenine dinucleotide phosphate (NADPH) (reduced coenzyme II)-dependent oxidoreductase that efficiently catalyzes the reduction of various cytotoxic carbonyl compounds in cells, such as oxidative stress products and various drugs. Moreover, AKR1B10 can reduce the highly reactive carbonyl group to a less toxic hydroxyl group, thereby protecting cells from carbonyl damage^3–6^. AKR1B10 also catalyzes the reduction of 13-cis retinal, 9-cis-retinal, and all-trans retinal to their corresponding retinols^7^.

Studies revealed the involvement of AKR1B10 in the development and carcinogenesis of some tumors through effects on tumor cell growth and survival. The enzyme is reported to be overexpressed in lung cancer (including lung squamous cell carcinoma and smoking-related lung adenocarcinoma), cholangiocarcinoma, oral squamous cell carcinoma, liver cancer, breast cancer, and pancreatic cancer^8–13^. Interestingly, AKR1B10 is downregulated in gastrointestinal cancer^14^. Abnormal AKR1B10 expression, such as its overexpression in lung cancer^15^, is currently considered a useful biomarker for the diagnosis and prognosis of some cancers.

At present, there is no report on the expression of AKR1B10 in LSCC. This study analyzed the expression of AKR1B10 in LSCC and the correlations of its expression with laryngeal carcinoma differentiation, tumor size, lymph node metastasis, and prognostic indices. Because the expression of Ki67, p53, and MMP2 has been confirmed to be related to invasion, prognosis, and lymph node metastasis in laryngeal carcinoma, we also analyzed the expression of these three proteins in laryngeal carcinoma, used statistical methods to analyze the correlation between AKR1B10 and these three proteins, and then analyzed whether AKR1B10 can regulate the expression of these three proteins. This may provide new ideas for further exploration of the occurrence, development, and prevention of LSCC.

## Results

### AKR1B10 is upregulated in laryngeal carcinoma and correlated with tumor size, lymph node metastasis, alcohol use, and differentiation

**Figure 1:**
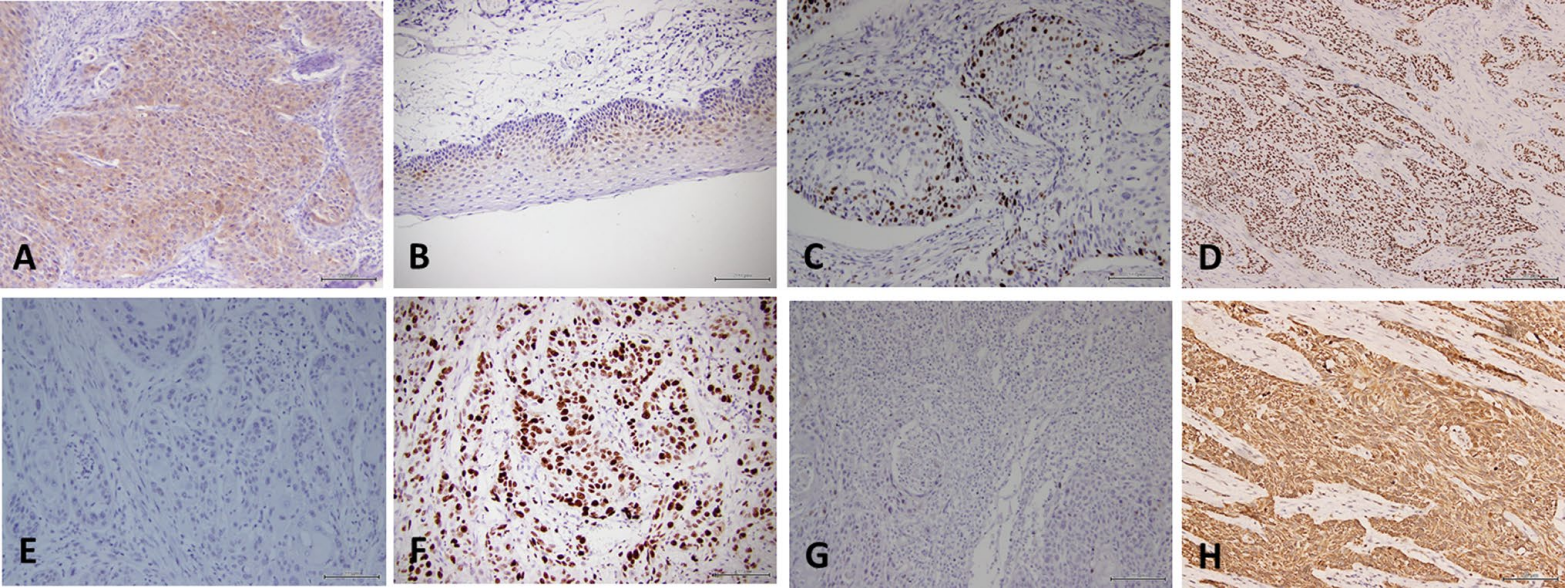
(**A**) Aldosterone reductase family 1 member B10 (AKR1B10) is expressed in the cytoplasm in laryngeal carcinoma tissues, and it is also expressed in individual nuclei. AKR1B10 was diffusely expressed in most laryngeal carcinoma cells. (**B**) AKR1B10 is expressed in the nuclei of cells in squamous epithelial tissue adjacent to the basal layer. (**C**) Low Ki-67 expression in laryngeal squamous cell carcinoma (LSCC). (**D**) High Ki-67 expression in LSCC. (**E**) Negative MTp53 expression in laryngeal carcinoma. (**F**) Positive MTp53 expression in laryngeal carcinoma. (**G**) Negative MMP2 expression in LSCC. (**H**) Positive MMP2 expression in LSCC.

AKR1B10 expression in laryngeal carcinoma cells was mainly localized in the cytoplasm, and the individual nuclei also displayed positive staining (Fig. 1A). Positive staining in the adjacent squamous epithelial tissues was mainly localized in the nuclei of cells near the basal layer (Fig. 1B). The expression pattern differed from that of laryngeal carcinoma. AKR1B10 expression was higher in LSCC tissues than in adjacent tissues (*P* < 0.001, Table 1). In addition, AKR1B10 is diffusely expressed in laryngeal carcinoma cells (Fig. 1A). However, Fig. 1B illustrates that AKR1B10 was only expressed in the area near the basal layer of the advanced squamous epithelium. AKR1B10 protein expression was higher in laryngeal carcinoma than in the adjacent squamous epithelium.

**Table 1:** Aldosterone reductase family 1 member B10 expression in laryngeal cancer and adjacent tissues.

Category	Total	Positive	Negative	Positive rate (%)	*P*
Laryngeal cancer	87	56	31	64.37	< 0.001
Paracancerous tissue	77	10	67	12.99	

The average age of the population was 55 years. AKR1B10 expression in laryngeal carcinoma was correlated with tumor size, lymph node metastasis, tumor differentiation, and alcohol use (all *P* < 0.05) but not with gender, age, T stage, and smoking status (Table 2).

**Table 2:** Relationships of tumor size, T stage, lymph node metastasis, tumor differentiation, age, gender, smoking status, and alcohol use with AKR1B10 expression in laryngeal carcinoma.

Clinical indicators	Number		AKR1B10				*P*
			−	+	+ +	+ + +	
Gender	Male	66	23	15	21	7	0.328
	Female	21	8	2	6	5	
Age (years)	≤ 60	50	22	10	10	8	0.062
	60	37	9	7	17	4	
Tumor size	≤ 2 cm	21	16	2	3	0	< 0.001
	> 2 cm	62	14	15	22	11	
T stage	T1–T2	63	27	19	8	9	0.632
	T3–T4	24	12	4	5	3	
Lymph node metastasis	Yes	39	8	8	16	7	0.01
	No	22	14	2	5	1	
Differentiation	High	19	10	5	3	1	0.007
	Medium	59	20	10	23	6	
	Poor	9	1	2	1	5	
Smoking	Yes	36	10	10	12	4	0.195
	No	51	21	13	7	10	
Alcohol use	Yes	25	2	6	8	9	< 0.001
	No	62	46	7	5	4	

+ , weakly positive; +  + , moderately positive; +  +  + , strongly positive.

All variables were analyzed using Fisher’s exact probability test, excluding age, which was analyzed using the R × C χ^2^ test.

AKR1B10, aldosterone reductase family 1 member B10.

### Expression of Ki-67 in laryngeal carcinoma

Ki-67 was positively expressed in the nuclei of laryngeal carcinoma cells. Ki-67 expression was high (Fig. 1D) in 55 cases (63.22%) and low (Fig. 1C) in 32 cases. Ki-67 expression was positively correlated with tumor size (r = 0.244, *P* < 0.05), alcohol use (r = 0.357, *P* < 0.01), and lymph node metastasis (r = 0.260, *P* < 0.05) and negatively correlated with the degree of differentiation (r =  − 0.483, *P* < 0.05, Table 3).

**Table 3:** Spearman’s correlation analysis of AKR1B10, Ki-67, MTp53, and MMP2 expression and clinical indicators.

Category	AKR1B10	MMP2	Ki-67 (%)	MTp53 (%)
Age	0.130	− 0.138	− 0.156	0.055
Gender	0.071	− 0.165	− 0.069	− 0.112
Tumor diameter	0.474**	0.235*	0.244*	0.196
Differentiation	− 0.322**	− 0.302**	− 0.483**	− 0.317**
T staging	0.082	− 0.127	− 0.174	0.070
Lymph node metastasis	0.394**	0.481**	0.260*	0.270*
Alcohol use	0.385**	0.376**	0.357**	0.374**
Smoking	0.142	0.159	0.053	0.045
MTp53 (%)	0.330**	0.226*	0.306**	
Ki-67 (%)	0.353**	0.387**		
MMP2	0.404**			

*AKR1B10* aldosterone reductase family 1 member B10, *MTp53* mutant p53, *MMP2* matrix metalloproteinase 2.

*P < 0.05 **P < 0.01.

### Expression of mutant p53 (MTp53) in laryngeal carcinoma and its relationship with clinical indicators

MTp53 was positively detected in the nuclei of laryngeal carcinoma tissues. Of 87 laryngeal carcinoma cases, 44 were positive for MTp53 (50.58%) (Fig. 1F). MTp53 expression in laryngeal carcinoma was positively correlated with lymph node metastasis (r = 0.270, *P* < 0.05), alcohol use (r = 0.374, *P* < 0.01), and negatively (Fig. 1E) correlated with the degree of differentiation (r =  − 0.317, *P* < 0.01, Table 3).

### Matrix metalloproteinase 2 (MMP2) expression in laryngeal carcinoma and its relationship with clinical indicators

MMP2 was mainly expressed in the cytoplasm and cell membrane in laryngeal carcinoma tissues. In total, 68 lesions were positive for MMP2 expression (78.16%, Fig. 1H). Its expression was positively correlated with tumor size (r = 0.235, *P* < 0.05), alcohol use (r = 0.376, *P* < 0.01), and lymph node metastasis (r = 0.481, *P* < 0.01) and negatively correlated with the degree of differentiation (r =  − 0.302, *P* < 0.01, Table 3).

### Correlation of AKR1B10 expression with Ki-67, MTp53, and MMP2 expression and clinical indicators

Immunohistochemistry revealed that among 87 laryngeal carcinoma tissues, 15 were AKR1B10^+^ Ki-67^high^, 14 were AKR1B10^−^ Ki-67^low^, 41 were AKR1B10^+^ Ki-67^low^, and 17 were AKR1B10^−^ Ki-67^high^. Spearman’s correlation analysis illustrated that AKR1B10 expression was positively correlated with Ki-67 expression (r = 0.353*, P* < 0.01, Table 3).

According to immunohistochemistry, 34 tissues were AKR1B10^+^ MTp53^+^, 21 were AKR1B10^−^ MTp53^−^, 22 were AKR1B10^+^ MTp53^−^, and 10 were AKR1B10^−^ MTp53^+^. Spearman’s correlation analysis indicated that AKR1B10 expression was positively correlated with MTp53 expression (r = 0.330, *P* < 0.01, Table 3).

Among 87 laryngeal carcinoma tissues, 51 were AKR1B10^+^ MMP2^+^, 14 were AKR1B10^−^ MMP2^−^, 5 were AKR1B10^+^ MMP2^−^, and 17 were AKR1B10^−^ MMP2^+^. Spearman’s correlation analysis revealed a positive correlation between AKR1B10 and MMP2 expression (r = 0.404, *P* < 0.01, Table 3).

### AKR1B10 mRNA expression in Hep-2 cells

**Figure 2:**
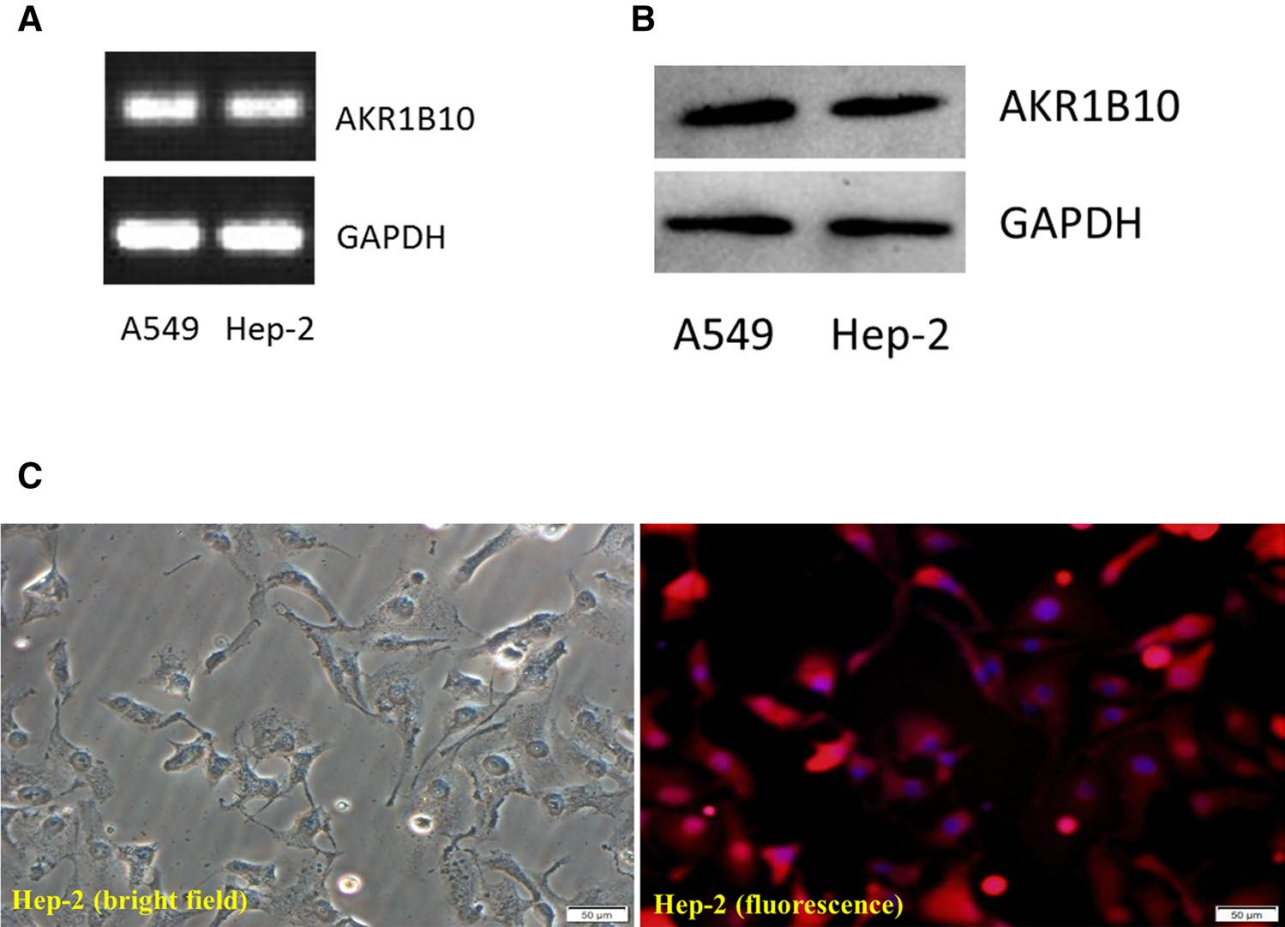
(**A**) Aldosterone reductase family 1 member B10 mRNA expression in Hep-2 cells. (**B**) Aldosterone reductase family 1 member B10 protein expression in Hep-2 cells. (**C**) Aldosterone reductase family 1 member B10 expression in Hep-2 cells (200×). Left panel, bright field; right panel, fluorescence.

RT A549 cells were used as positive controls, and Hep-2 cells served as the experimental group. Hep-2 cells were found to express AKR1B10 mRNA (Fig. 2A).

### AKR1B10 protein expression in Hep-2 cells

Western blotting demonstrated that compared with A549 cells, Hep-2 cells expressed AKR1B10 protein (Fig. 2B).

Immunofluorescence staining illustrated that AKR1B10 was mainly expressed in the cytoplasm of Hep-2 cells, and positive expression was also observed in individual nuclei (Fig. 2C).

### Effects of oleanolic acid (OA) on AKR1B10 enzyme activity in Hep-2 cells

**Figure 3:**
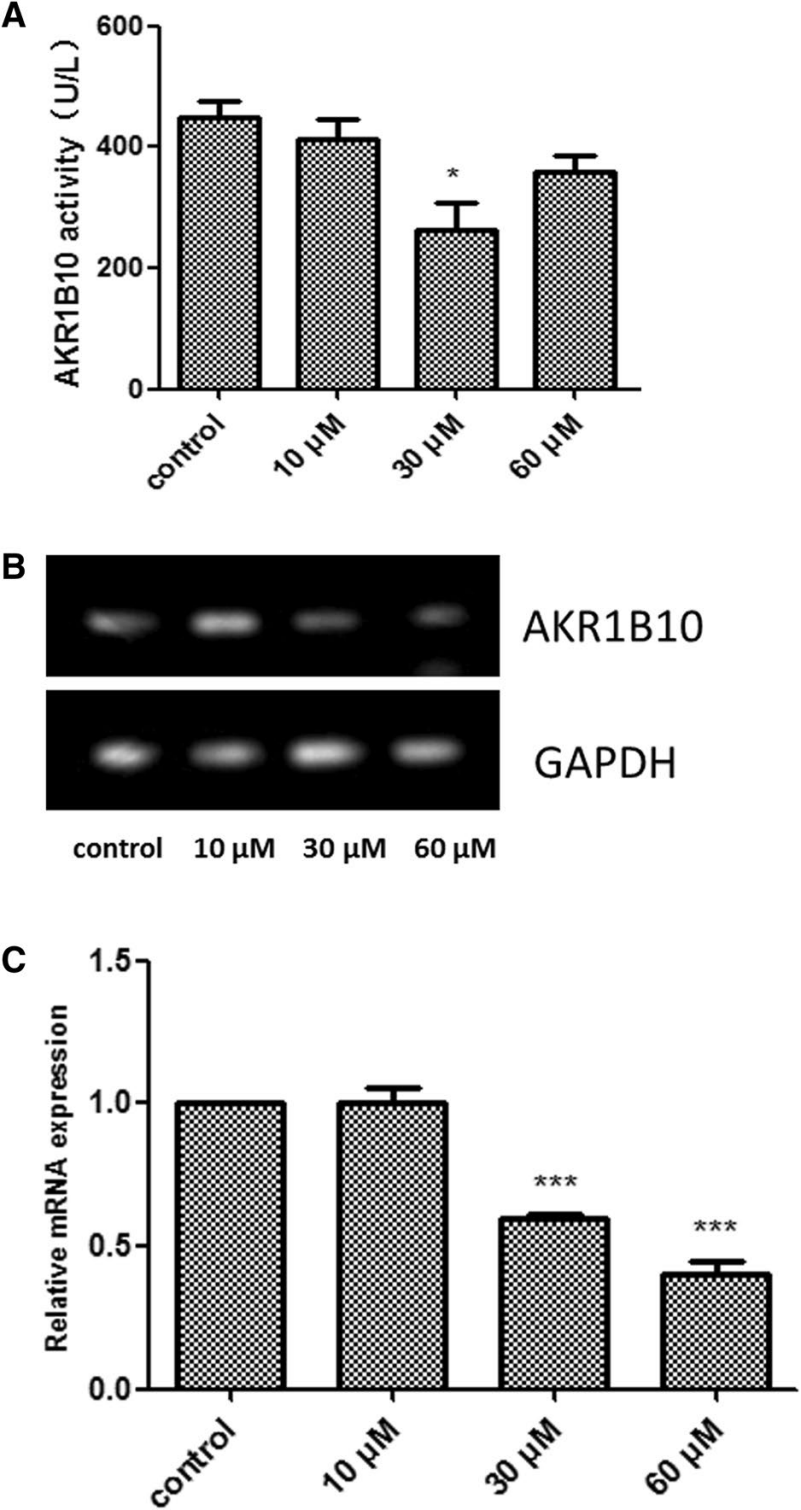
(**A**) Effects of oleanolic acid on aldosterone reductase family 1 member B10 (AKR1B10) enzyme activity. *P < 0.05, compared with the control group. (**B**) Aldosterone reductase family 1 member B10 mRNA expression in Hep-2 cells following treatment with oleanolic acid. (**C**) Quantification of aldosterone reductase family 1 member B10 (AKR1B10) mRNA expression in Hep-2 cells following treatment with oleanolic acid. ***P < 0.001 compared with the control group.

Compared with the findings in the control group, AKR1B10 activity in Hep-2 cells was significantly decreased after 48 h of treatment with 30 μM OA (*P* < 0.05, Fig. 3A). Conversely, the decrease was not significant after exposure to 10 or 60 μM.

### Effects of OA on AKR1B10 mRNA expression in Hep-2 cells

Reverse-transcription polymerase chain reaction (RT-PCR) was used to detect the effects of 48 h of OA exposure on AKR1B10 mRNA expression in Hep-2 cells. Compared with the control group, the 30 and 60 μM OA groups significantly inhibited the mRNA expression of AKR1B10 (both *P* < 0.01, Fig. 3B,C).

### Effects of OA on AKR1B10 protein expression in Hep-2 cells

**Figure 4:**
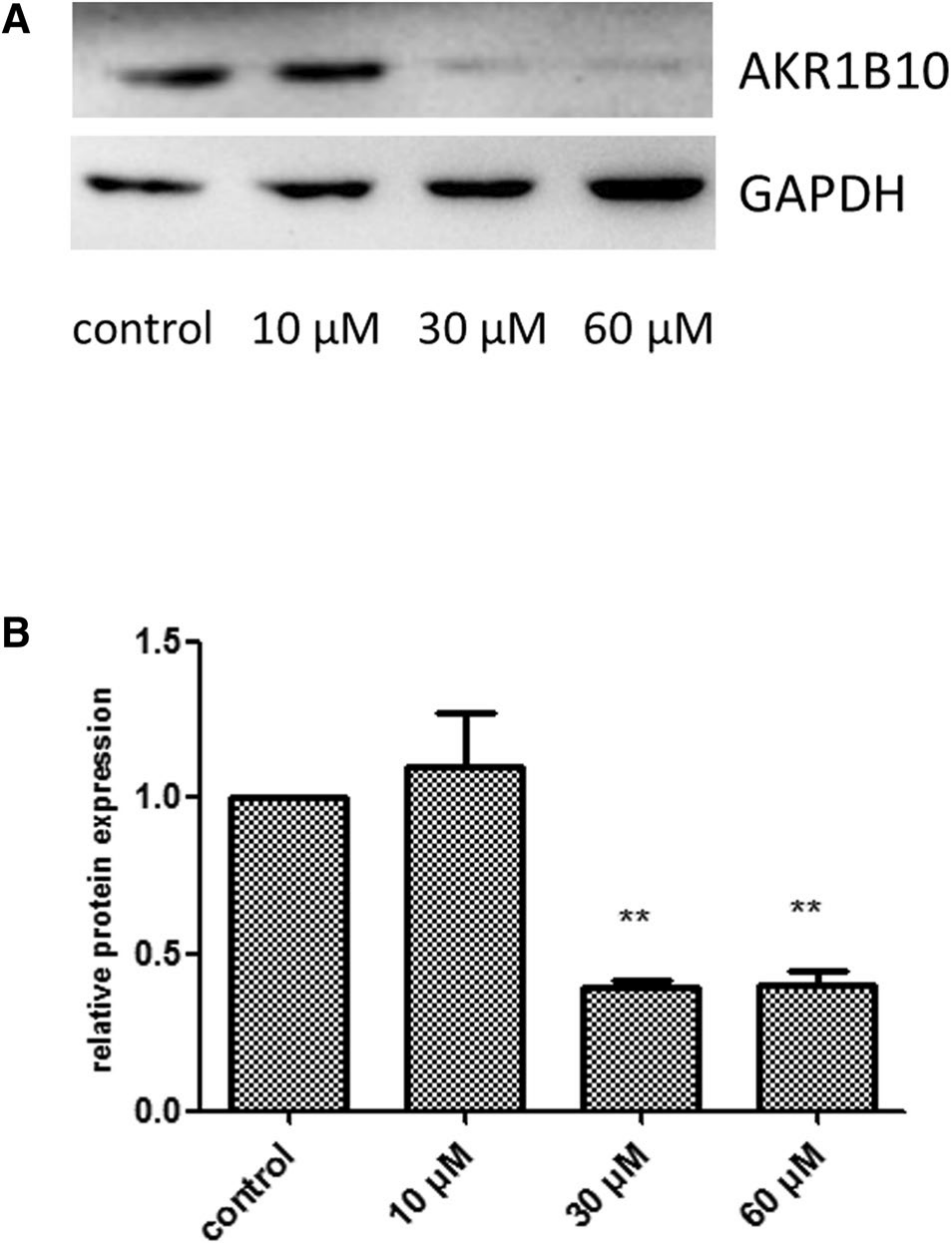
(**A**) Aldosterone reductase family 1 member B10 (AKR1B10) protein expression in Hep-2 cells treated with oleanolic acid. (**B**) Quantification of aldosterone reductase family 1 member B10 AKR1B10 protein expression in Hep-2 cells treated with oleanolic acid. **P < 0.01, compared with the control group.

Compared with the control group, the 30 and 60 μM OA groups significantly inhibited the protein expression of AKR1B10 (*P* < 0.01, Fig. 4A,B).

### Effects of OA on the proliferation of Hep-2 cells

**Figure 5:**
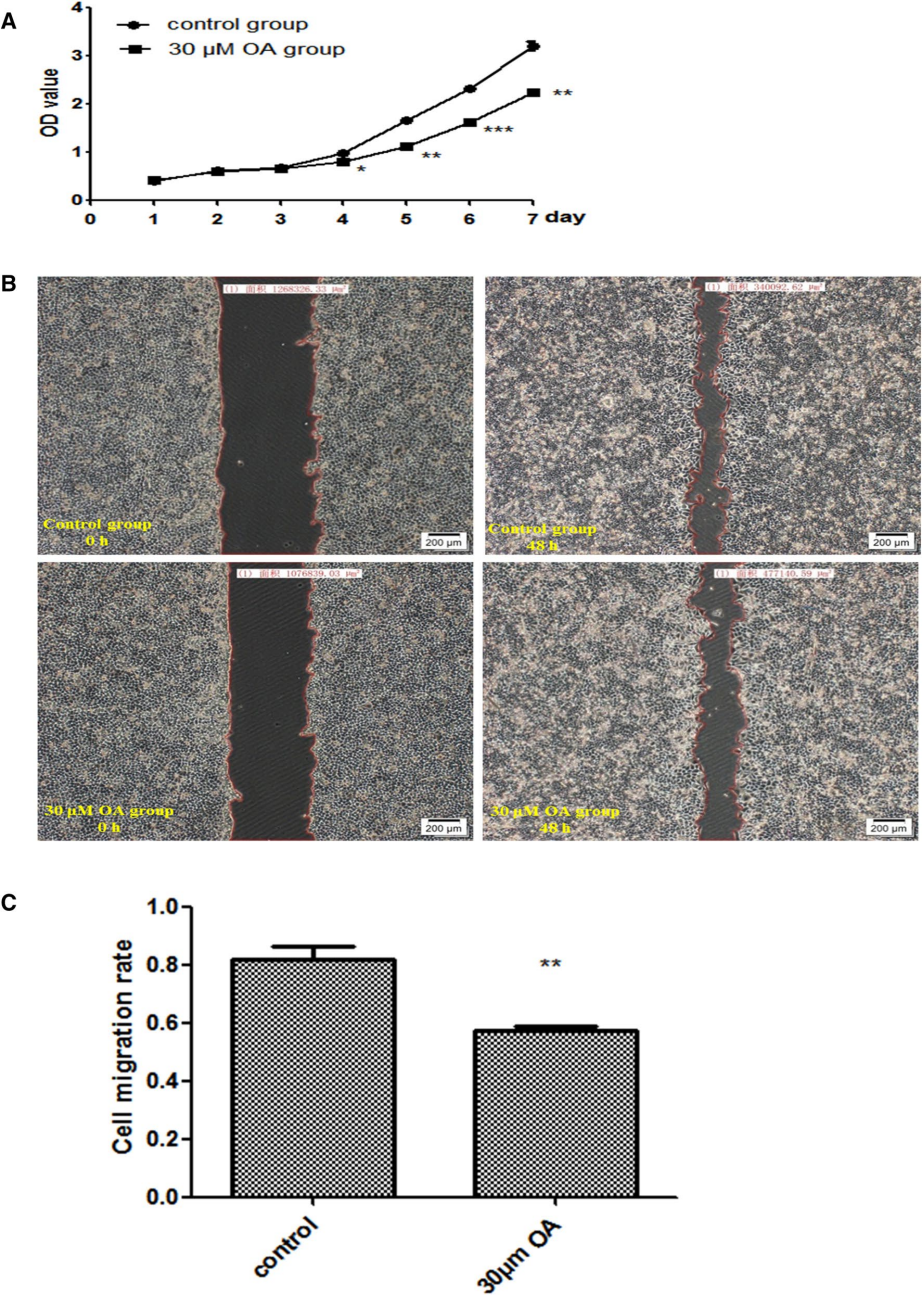
(**A**) Proliferation of Hep-2 cells following treatment with oleanolic acid (OA). *P < 0.05, **P < 0.01, ***P < 0.001, compared with the control group. (**B**) Changes in the migration of Hep-2 cells following treatment with oleanolic acid (40 ×). Upper left panel, Control group 0 h; upper right panel., Control group 48 h; bottom left panel, 30 μM OA group 0 h; bottom right panel, 0 μM OA group 48 h. (**C**) Quantification of the migration of Hep-2 cells following treatment with OA. **P < 0.01, compared with the control group.

The results of the CCK8 assay demonstrated that the 30 μM OA group significantly decreased Hep-2 cell growth starting on day 4 as compared to the control group (*P* < 0.05 on day 4, *P* < 0.01 on day 5, *P* < 0.001 on day 6, and *P* < 0.01 on day 7; Fig. 5A).

### Effect of OA on the migration of Hep-2 cells

The Hep-2 cells were treated with 0 or 30 μM OA for 48 h to observe changes in cell migration. Cell mobility was somewhat decreased by 30 μM OA compared with the control group findings (*P* < 0.01, Fig. 5B,C).

### Effects of OA on the invasiveness of Hep-2 cells

**Figure 6:**
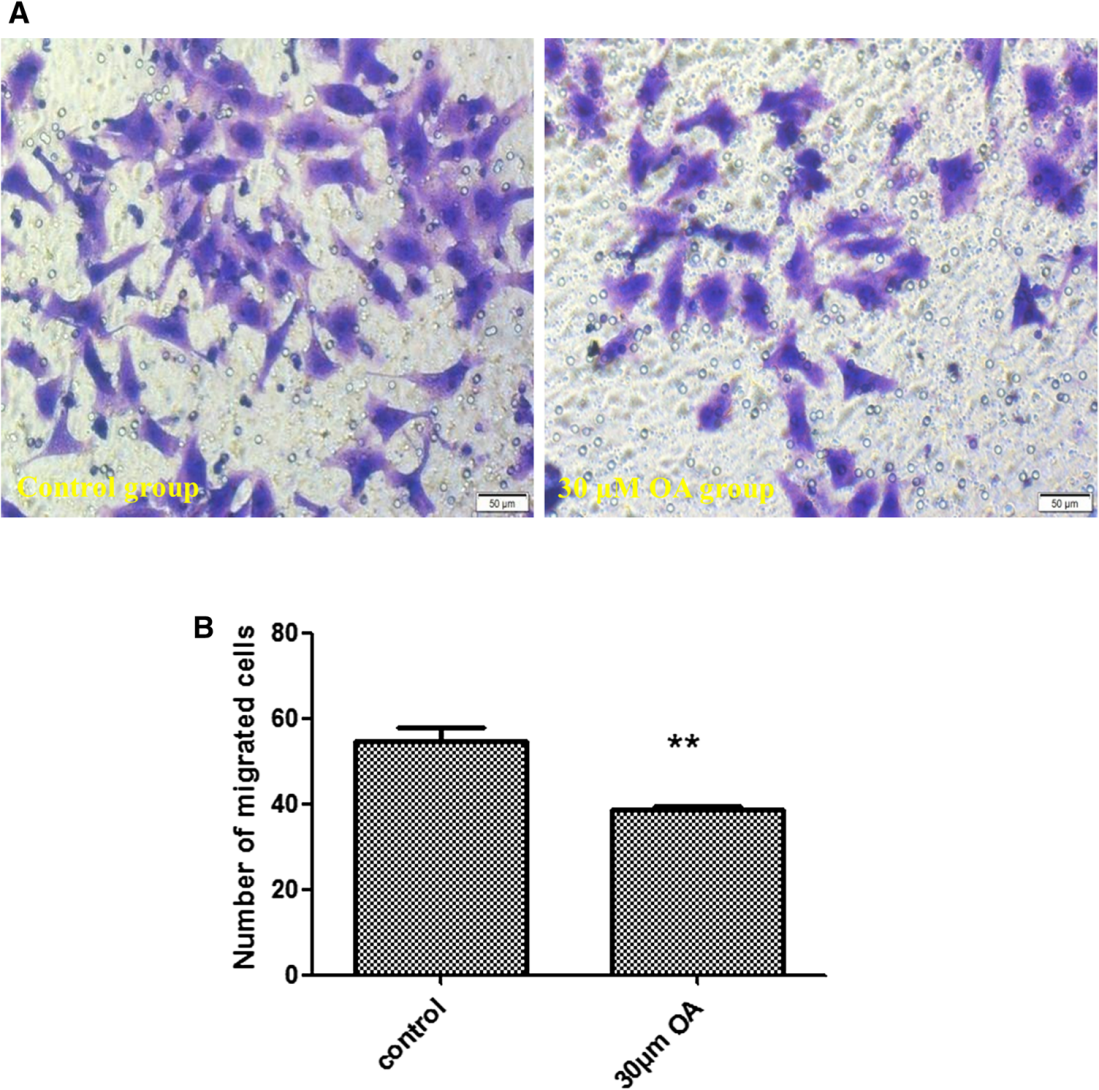
(**A**) Invasiveness of Hep-2 cells after 48 h of oleanolic acid (OA) exposure (200×). Left panel, Control group; right panel, 30 μM OA group). (**B**) Quantification of the invasiveness of Hep-2 cells following treatment with OA. **P < 0.01, compared with the control group.

According to the Transwell assay, 30 μM OA significantly suppressed the invasiveness of Hep-2 cells compared with the control findings (*P* < 0.01, Fig. 6A,B).

## Discussion

Globally, LSCC accounts for almost 2% of all malignancies, and it is one of the most common tumors of the head and neck^16^. AKR1B10 was initially isolated from liver cancer lesions^2^. Matkowskyj et al. found that AKR1B10 is upregulated in hepatocarcinoma, and silencing of the AKR1B10 gene in liver cancer cells increased apoptosis and reduced the formation and size of tumor cell colonies. This study provided the first evidence that AKR1B10 participates in hepatocellular carcinogenesis by regulating cell proliferation and apoptosis, and it can be used to identify hepatocellular carcinoma and benign liver lesions. In addition, the enzyme is an independent biomarker^17^. Fukumoto et al.^10^ found that AKR1B10 is overexpressed in non-small cell lung cancer and is closely related to smoking. Ma et al.^13^ reported that AKR1B10 overexpression was associated with tumor size, lymph node metastasis, and patient survival in breast cancer. Chung et al.^8^ found that AKR1B10 is overexpressed in pancreatic cancer, and it may participate in carcinogenesis by regulating apoptosis and protein prenylation. However, Ohashi et al.^14^ demonstrated that AKR1B10 is downregulated in gastrointestinal cancer. AKR1B10 is highly expressed in normal gastrointestinal tissues. It reduces toxic substances produced by diet and microbial metabolism in the gastrointestinal tract via carbonyl detoxification, and it can eliminate oxidative stress and promote epithelial cell proliferation. It is an important protective factor for gastrointestinal epithelial cells. AKR1B10 downregulation or ablation is conducive to the occurrence of cancer^18^. However, studies on AKR1B10 expression in laryngeal carcinoma have not been reported. This experiment was the first to detect the expression of AKR1B10 in laryngeal and precancerous tissues via immunohistochemistry, and the relationships of AKR1B10 with differentiation, tumor size, lymph node metastasis, and Ki-67, MTp53, and MMP2 expression were analyzed. In addition, the effects of AKR1B10 on the biological behavior of Hep-2 cells were detected via in vitro experiments, and the significance of its expression in laryngeal carcinoma was further explored.

The findings of the study were consistent with a study by Yoshitake et al.^19^, who found that AKR1B10 is highly expressed in the cytoplasm or nuclei of cervical cancer cells and occasionally expressed in normal squamous epithelial tissues, wherein it exhibits nucleus-specific expression. We hypothesized that AKR1B10 overexpression may be involved in the development and progression of laryngeal cancer. Kang et al.^20^ reported that AKR1B10 is upregulated in lung squamous cell carcinoma, and AKR1B10 mRNA expression in highly differentiated tumors was significantly lower than that in moderately poorly differentiated tumors, in line with our results. However, it has also been reported that AKR1B10 is overexpressed in nasopharyngeal carcinoma, and the expression in moderately well differentiated tumors is higher than that in poorly differentiated tumors^21^. This may be attributable to differences in the role of AKR1B10 in different tumors. The finding that AKR1B10 expression was correlated with tumor size was consistent with the previously reported expression of AKR1B10 in oral squamous cell carcinoma and breast cancer^11,13^. In addition, Ma et al.^13^ found that AKR1B10 is overexpressed in breast cancer and is positively associated with lymph node metastasis, as demonstrated in the present analysis. However, studies revealed that AKR1B10 expression in oral squamous cell carcinoma and cervical cancer is not associated with lymph node metastasis^11,22^.

Although AKR1B10 is upregulated in oral squamous cell carcinoma, breast cancer, cervical cancer, and lung cancer, the relationships of AKR1B10 expression with tumor size, lymph node metastasis, and squamous cell carcinoma differentiation are different^11,13,22,23^. In addition, studies revealed that AKR1B10 is overexpressed in breast cancer, oral squamous cell carcinoma, cervical cancer, and other tumors and is associated with prognosis^11,13,22^. To further analyze whether AKR1B10 expression in laryngeal carcinoma also has prognostic significance, we analyzed the relationships of AKR1B10 with the prognostic indicators Ki-67, MTp53, and MMP2.

The nuclear protein Ki-67 is closely related to tumor cell proliferation, and its overexpression is positively correlated with tumor malignancy. Ki-67 is a prognostic marker for breast cancer, lung cancer, cervical cancer, and other tumors^22^. Gioacchini et al.^23^ demonstrated that Ki-67 is overexpressed in laryngeal carcinoma and is associated with poor tumor cell invasion and prognosis. It has been reported that the expression of Ki-67 in highly differentiated laryngeal carcinoma is significantly lower than that in poorly differentiated laryngeal carcinoma^24^. The expression rate of Ki-67 in laryngeal carcinoma with lymph node metastasis is significantly higher than that in laryngeal carcinoma without lymph node metastasis^25^. Previously, Wei et al.^26^ silenced AKR1B10 in MHCC97H liver cancer cells, finding that Ki-67 and the oncogenes c-myc, c-fos, and N-ras were downregulated and the pro-apoptotic protein genes caspase-3 and Bax were upregulated, indicating that AKR1B10 may promote cell proliferation, inhibit cell apoptosis, and induce hepatocyte deterioration by regulating the expression of tumor-associated genes. However, Schmitz et al.^27^ reported that the proliferative capacity of AKR1B10-positive hepatocellular carcinoma and its Ki-67 expression rate were significantly lower than those of AKR1B10-negative hepatocellular carcinoma. However, our study only found a positive correlation between AKR1B10 and Ki-67 expression. The specific links among these factors remain to be elucidated.

The tumor suppressor gene p53 protein is lost or mutated in approximately half of human cancers. It is well known that the loss of p53 function affects cell proliferation, migration, and invasion^28^. Wang et al. found that the 5-year survival rate of patients with LSCC and high MTp53 expression was significantly lower than that of patients with LSCC and low MTp53 expression, suggesting that MTp53 is associated with prognosis^29^. Our findings regarding MTp53 were consistent with the results of Ji et al., who reported that MTp53 expression in oral squamous cell carcinoma was associated with lymph node metastasis and tumor differentiation, but not age, gender, or tumor location^30^. Therefore, we speculate that AKR1B10 could be an indicator of poor prognosis in laryngeal cancer.

MMP2 is a gelatinase that degrades basement membrane collagen, which is related to the metastasis of various human tumors^31^. Liu et al. found that MMP2 is highly expressed in laryngeal carcinoma, and its expression in highly differentiated tumors is significantly lower than that in moderately poorly differentiated tumors. Additionally, MMP2 expression in lymph node metastasis is significantly higher than that in non-metastatic tissues. This indicates that MMP2 may be involved in the development and prognosis of laryngeal cancer^32^. Our results are consistent with this supposition. In particular, our results were similar to those of Li et al., who found that AKR1B10 expression is positively correlated with tumor size and lymph node metastasis in breast cancer and that AKR1B10 promotes MMP2 and vimentin expression by activating the ERK signaling pathway, thereby promoting the invasion and metastasis of MCF-7 and BT-20 breast cancer cells^33^. Whether AKR1B10 directly or indirectly promotes the occurrence and metastasis of laryngeal cancer through MMP2 remains to be further studied.

In vitro experiments were performed to confirm the aforementioned findings using the highly selective AKR1B10 inhibitor OA^34^ and Hep-2 cells. Our results were consistent with the findings of Zhou et al., who demonstrated that silencing of the AKR1B10 gene inhibited lung cancer cell proliferation and migration^35^. Our findings regarding the inhibitory effects of OA on AKR1B10 expression and activity were consistent with the aforementioned immunohistochemistry results. Therefore, we speculate that the overexpression of AKR1B10 in laryngeal carcinoma may be related to the occurrence and poor prognosis of cancer.

Certain limitations of our work must be addressed. We did not perform a quantitative analysis of AKR1B10 expression at the tissue level. Additional experiments are needed to further clarify the mechanism by which AKR1B10 affects the phenotype of LSCC. We plan to perform in vivo experiments to further study the role of AKR1B10 in laryngeal cancer in the future.

This study was the first to detect the expression of AKR1B10 in laryngeal carcinoma and explore its relationship with tumor differentiation, tumor size, lymph node metastasis and prognosis. Based on the findings, AKR1B10 may represent a new target and theoretical molecular basis for the treatment and prognosis of laryngeal cancer.

## Methods

### Patient samples and cell culture

In total, 87 cases of LSCC detected between 2015 and 2017 in the second department of Jilin University First Hospital were examined in the study. The female to male ratio was 3.14:1. Sixty-one cases involved lymph node metastasis (Table 4), and 77 cases involved invasion of adjacent tissues. This study was approved by the First Hospital of Jilin University’s Institutional Review Board. All methods were performed in accordance with relevant guidelines and regulations. Informed consent was obtained from all patients.

**Table 4:** Laryngeal cancer specimen information.

Clinical index	Number
**Gender**	
Male	66
Female	21
Age (years), range	40–80
≤ 60 years old	50
> 60 years old	37
**Tumor differentiation**	
High	19
Medium	59
Poor	9
**T stage**	
T1–T2	63
T2–T3	24
**Lymph node metastasis**	
Yes	39
No	22
**Tumor size**	
≤ 2 cm	21
> 2 cm	62
**Smoking**	
Yes	36
No	51
**Alcohol use**	
Yes	25
No	62

Of the 87 cases of laryngeal cancer, 26 did not feature lymph nodes metastasis, whereas the tumor size could not be determined in 4 cases.

Hep-2 and A549 cells^36^ were provided by the Key Laboratory of Pathology and Biology of the Ministry of Education of Jilin University. The cells were cultured in H-DMEM containing 10% fetal bovine serum in an incubator containing 5% CO_2_ at 37 °C.

### Immunohistochemistry

Immunohistochemical staining was performed to analyze the expression of AKR1B10, Ki-67, MTp53, and MMP2 in LSCC and adjacent tissues. Tissue sections were incubated at 70 °C for 1 h, followed by incubation with 100% xylene for 10 min, 100% ethanol twice for 5 min each, and then 95%, 90%, and 80% ethanol for 2 min each. The slides were washed and repaired under high pressure. After setting up and running the automatic immunohistochemical stainer program, slides were counterstained, differentiated, subjected to anti-blue staining, dehydrated, and cleared. Slides were finally observed under a microscope.

### Result interpretation

AKR1B10 and MMP2 staining intensity was scored as the percentage of positive cells as follows: < 5%, 0 points; 5–10%, 1 point; 11–50%, 2 points; and > 50%, 3 points Meanwhile, the staining color was scored as follows: no color, 0 points; light yellow, 1 point; dark yellow, 2 points; and yellowish brown, 3 points. The staining score was calculated by summing the intensity and color scores and categorized as follows: < 2, negative; 2–3, weakly positive; 4–5, moderately positive; and ≥ 6, strongly positive^17^.

For Ki-67 scoring, the percentage of positive cells among 1000 positive cells was categorized as follows: ≤ 25%, low expression; and > 25%, high expression^37^.

MTp53 expression was scored as the percentage of positive cells among 1000 cells as follows: ≤ 10%, negative; and > 10%, positive^38^.

### Reverse-transcription polymerase chain reaction (RT-PCR)

Ribonucleic acid (RNA) was extracted from Hep-2 and A549 cells. RNA was reverse-transcribed into cDNA according to the instructions of the kit. After the PCR reaction system was thoroughly mixed, 30 cycles were performed according to the reaction conditions.

PCR reaction system.

**Table:** 

Reagent	Volume (μL)
cDNA	1
Upstream primer	0.4
Downstream primer	0.4
SuperMix	10
ddH_2_O	8.2
Total system volume	20

PCR reaction conditions.

**Table:** 

Reaction conditions (°C)	Time
94	5 min
94	30 s
60	30 s
72	1–2 kb/min
72	10 min

For electrophoresis, 10 μL of the PCR product were added to each well and electrophoresed at 100 V for 1 h, and the results were observed using an imager.

### Western blotting

To extract protein, Hep-2 and A549 cells were washed with cold phosphate-buffered saline (PBS), after which the culture dishes were placed on ice. Cells were lysed with 500 μL of lysate per dish for 30 min, and cells were scraped into Eppendorf tubes and centrifuged at 15 × 10 rpm for 15 min. The supernatant was retained and used to measure the protein concentrations in the samples.

For electrophoresis, 30 μg of the protein sample were added to each well, electrophoresed at 80 V for 30 min, and then electrophoresed at 120 V for 90 min. Proteins were then transferred to a membrane and incubated sequentially with primary and secondary antibodies. Due to the need to add different antibodies during the experiment, the PDVF membrane was cut.

### Immunofluorescence staining

Hep-2 and A549 cells were cultured in 24-well plates. After permitting adherent growth, cells were fixed with paraformaldehyde for 20 min, washed three times with PBS, incubated with 0.3% Triton for 5 min, and washed three times with PBS. Cells were then exposed to 0.5% BSA for 30 min. After aspirating the liquid, cells were incubated with 0.1% BSA-diluted AKR1B10 antibody (1:50) overnight at 4 °C and washed three times with PBS. Cells were incubated with the fluorescent secondary antibody (1:400) at room temperature for 1 h, washed three times with PBS, stained with DAPI for 10 min, and washed with PBS, and the stained cells were observed under a fluorescence microscope.

### Effect of OA on the activity and expression of AKR1B10 in Hep-2 cells

To conduct ELISA, 6 × 10^5^ Hep-2 cells were added to each well of a six-well plate. After permitting adherent growth, cells were cultured with 0, 10, 30, or 60 μM OA for 48 h and lysed (approximately 1 million cells/ml). Cells were then centrifuged (3 × 1000 rpm, 10 min). According to the AKR1B10 enzyme activity assay kit instructions, a standard curve was drawn to calculate enzyme activity. For RT-PCR and Western blotting, cells were treated with OA, after which RNA and protein were extracted. AKR1B10 mRNA and protein levels in cells were detected via RT-PCR and Western blot, respectively.

Based on the results of the aforementioned experiments, 30 μM OA was selected for the subsequent experiments.

### CCK8 experiment

Hep-2 cells were cultured in seven 96-well plates at 2000 cells/100 μL per well. Three replicates each were created for the control and experimental groups. The control group was treated with H-DMEM containing 10% serum. The experimental group was treated with H-DMEM containing 30 μM OA. Cell growth was measured continuously for 7 days by adding 10 μL of CCK8 solution at 37 °C for 1–4 h, measuring the absorbance at 450 nm, and plotting the proliferation curve.

### Scratch test

A straight line was drawn on the back of a six-well plate. Hep-2 cells (6.5 × 10^5^) were added to each well and cultured overnight. Then, the cell monolayer was scratched in a direction perpendicular to the line. The control group was cultured in serum-free H-DMEM. The experimental group was cultured in serum-free H-DMEM containing 30 μM OA. Cell migration was observed under a phase contrast microscope at 0 and 48 h. Cell migration was calculated using the following formula:

$$ {\text{Relative}}\;{\text{mobility}}\;{\text{of}}\;{\text{cells}} = \frac{A - B}{A}, $$

where A is the area of the scratch at 0 h and B is the area of the scratch at 48 h.

### Transwell experiment

Initially, 65 μL of diluted Matrigel were added in the upper chamber (Matrigel:H-DMEM ratio = 1:5). Then, Hep-2 cells (7 × 10^4^ cells/200 μL) cultured in serum-free H-DMEM for 12 h were added to the upper chamber. The experimental group was cultured in H-DMEM containing 30 μM OA, and the control group was cultured in H-DMEM. Meanwhile, 500 μL of H-DMEM medium containing 15% serum were added to the lower chamber in both groups. Cells were incubated for 48 h in a 5% CO_2_ incubator at 37 °C. The culture medium in the upper chamber was discarded, and the cells in the upper chamber were cleaned with a cotton swab and fixed with paraformaldehyde for 20 min. After staining with crystal violet, the cells were observed under an inverted microscope.

### Statistical analysis

AKR1B10 expression in laryngeal cancer and paracancerous tissues was compared using *t*-test. Paracancerous tissue refers to tissue located within 2 cm of cancer tissue. The relationships of clinical indicators, excluding age, with AKR1B10 expression in laryngeal carcinoma was analyzed using Fisher’s exact probability test, whereas the correlation between age and AKR1B10 expression was analyzed using the R × C χ^2^ test. Nonsmokers comprised never-smokers and people with a past history of light smoking (< 10-pack–year history with at least 20 years of cessation). Alcohol use was defined as the consumption of 10–20 alcohol drinks weekly. Spearman’s correlation analysis was used to analyze the correlations of AKR1B10, Ki-67, MTp53, and MMP2 expression and clinical indicators.

## Supplementary Information

Supplementary Information.

## Abbreviations

LSCC: Laryngeal squamous cell carcinoma
OA: Oleanolic acid
DAPI: 4′,6-Diamidino-2-phenylindole
NADPH: Nicotinamide adenine dinucleotide phosphate
PBS: Phosphate-buffered saline
PCR: Polymerase chain reaction
RNA: Ribonucleic acid
