# Supplementary material for: The expression and significance of AKR1B10 in laryngeal squamous cell carcinoma
Source: Sci Rep. 2021 Sep 14;11:18228. doi: 10.1038/s41598-021-97648-y (PMC8440551; doi:10.1038/s41598-021-97648-y)
Supplement: Supplementary file 1 — Supplementary Information. [file 41598_2021_97648_MOESM1_ESM.pdf]

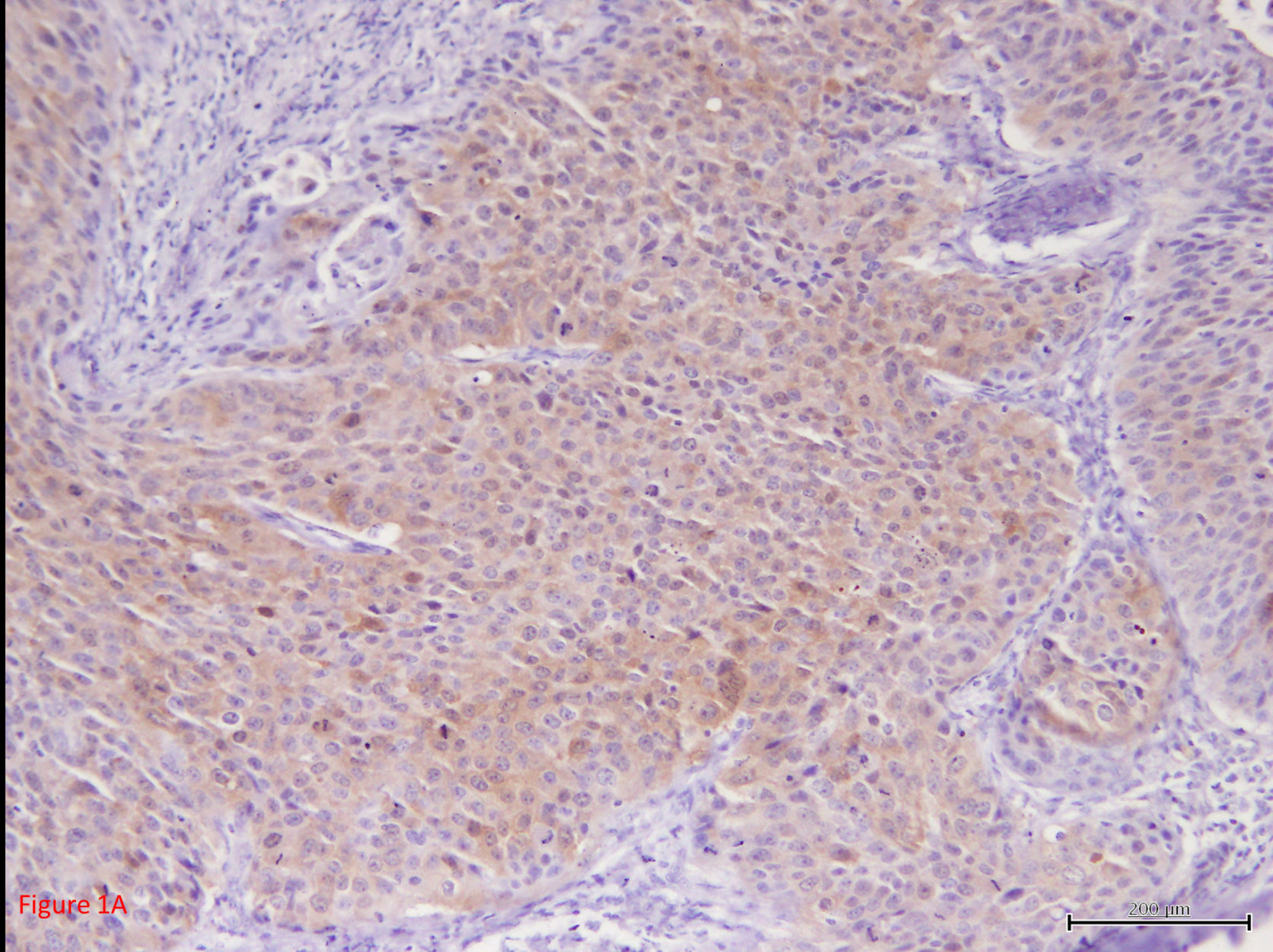

Figure 1A

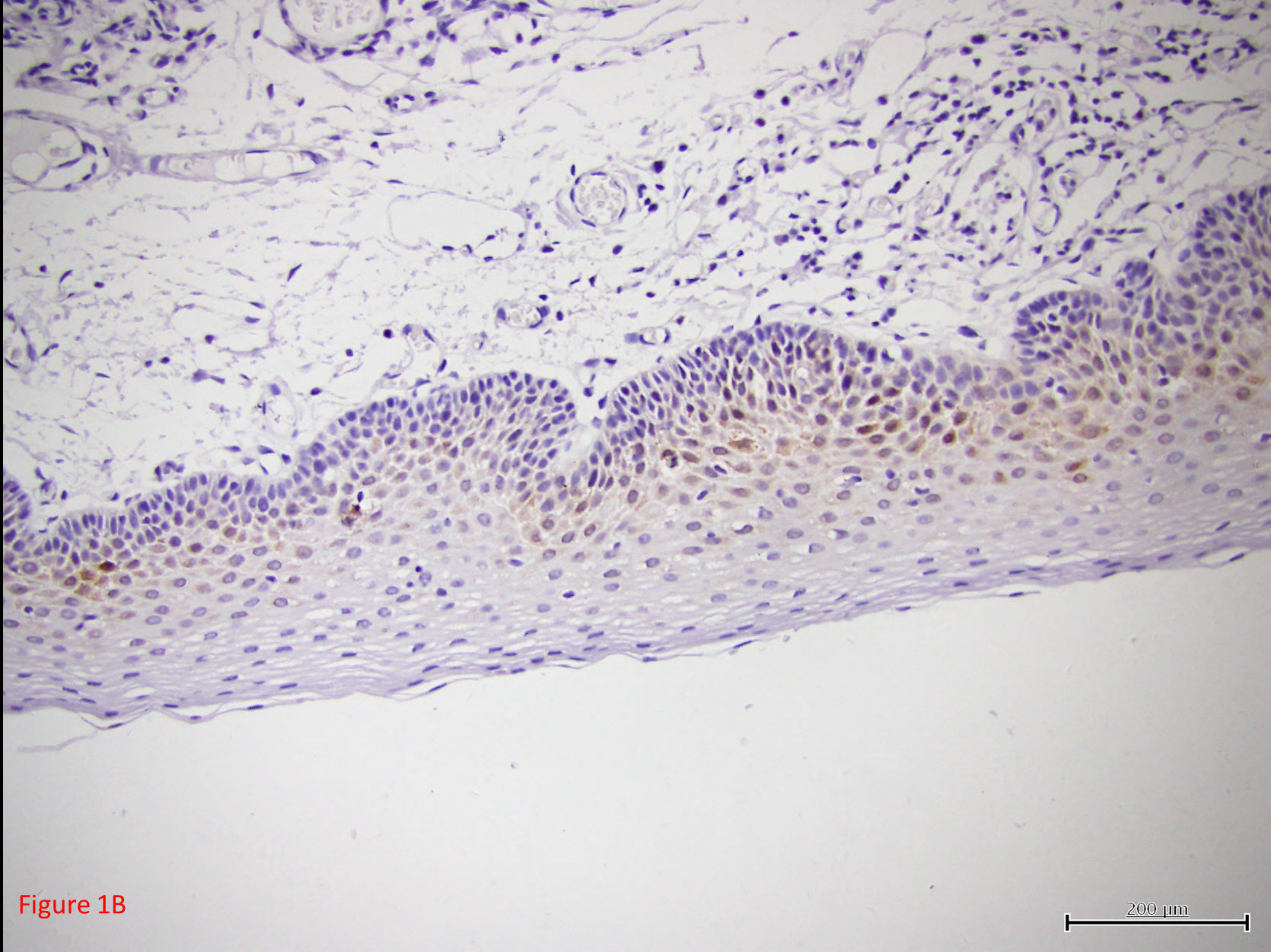

Figure 1B

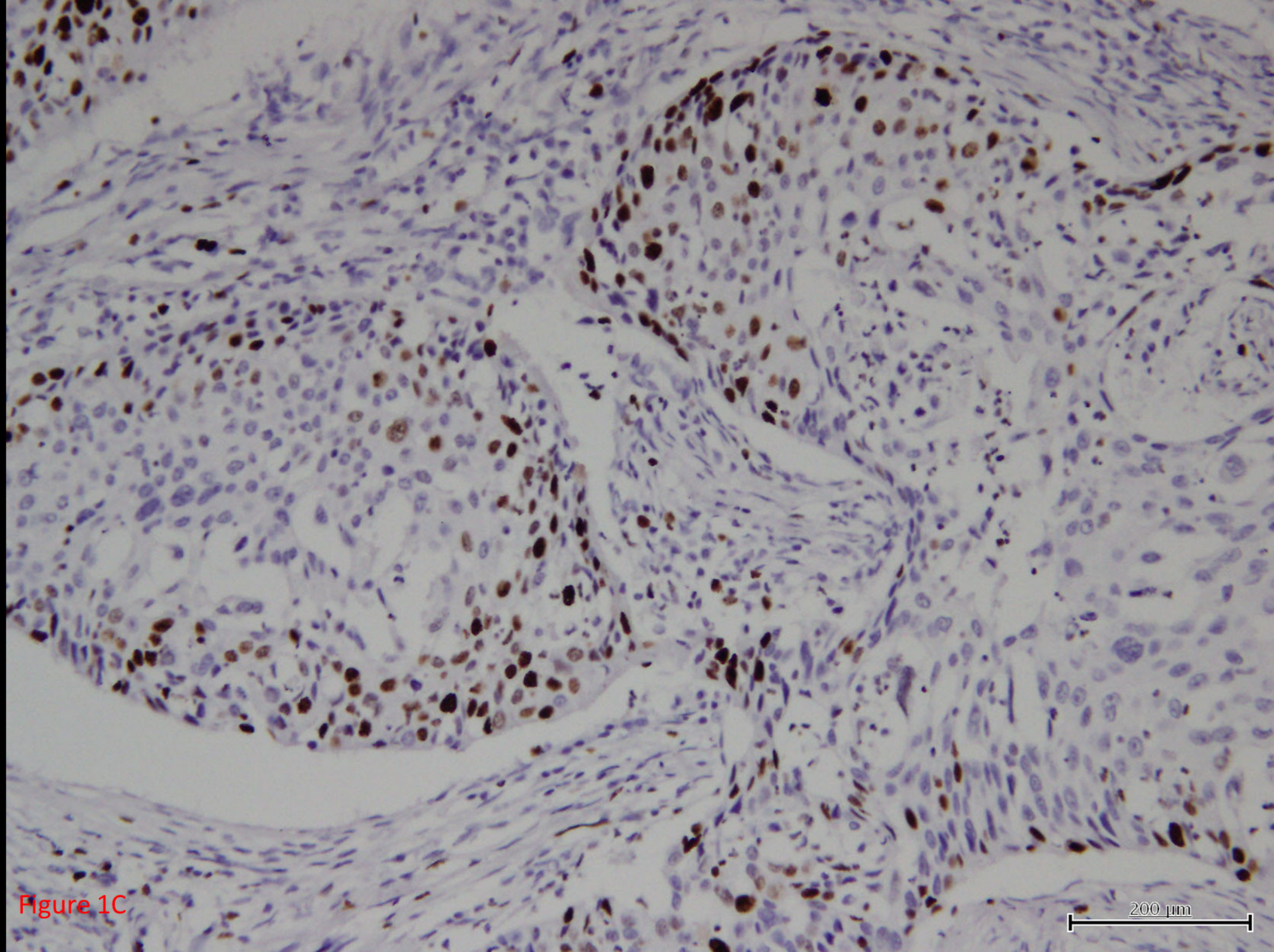

Figure 1C

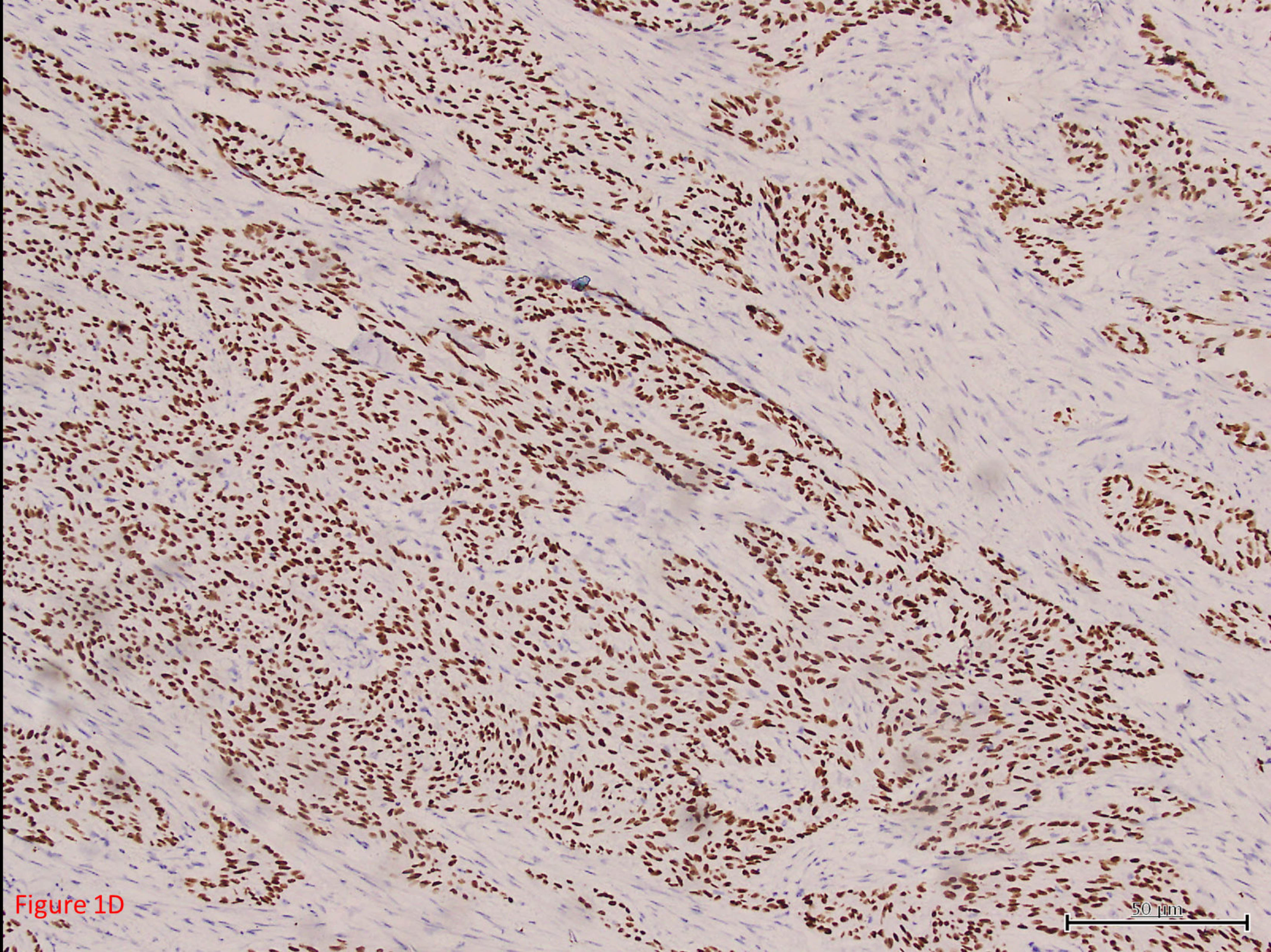

Figure 1D

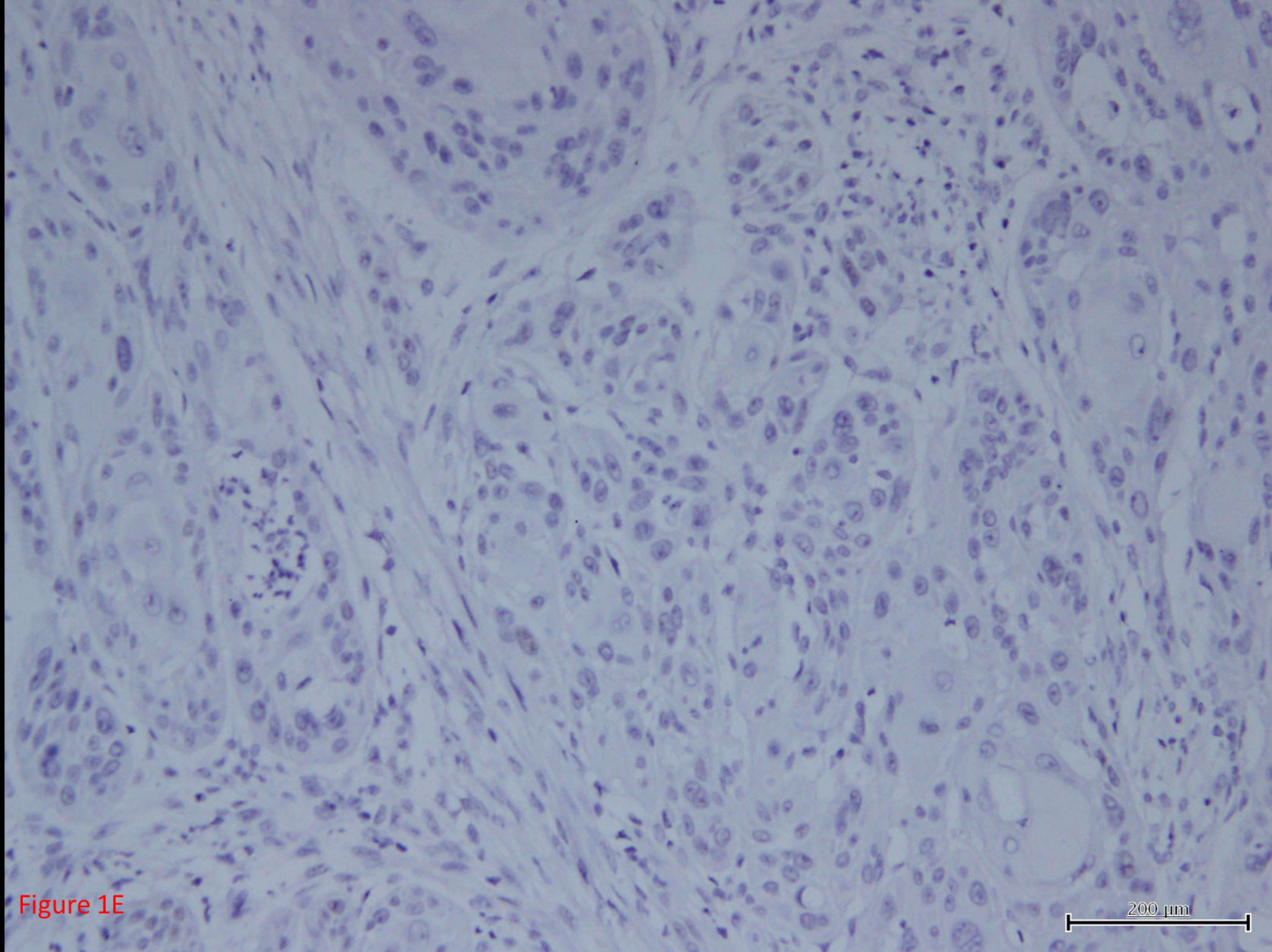

Figure 1E

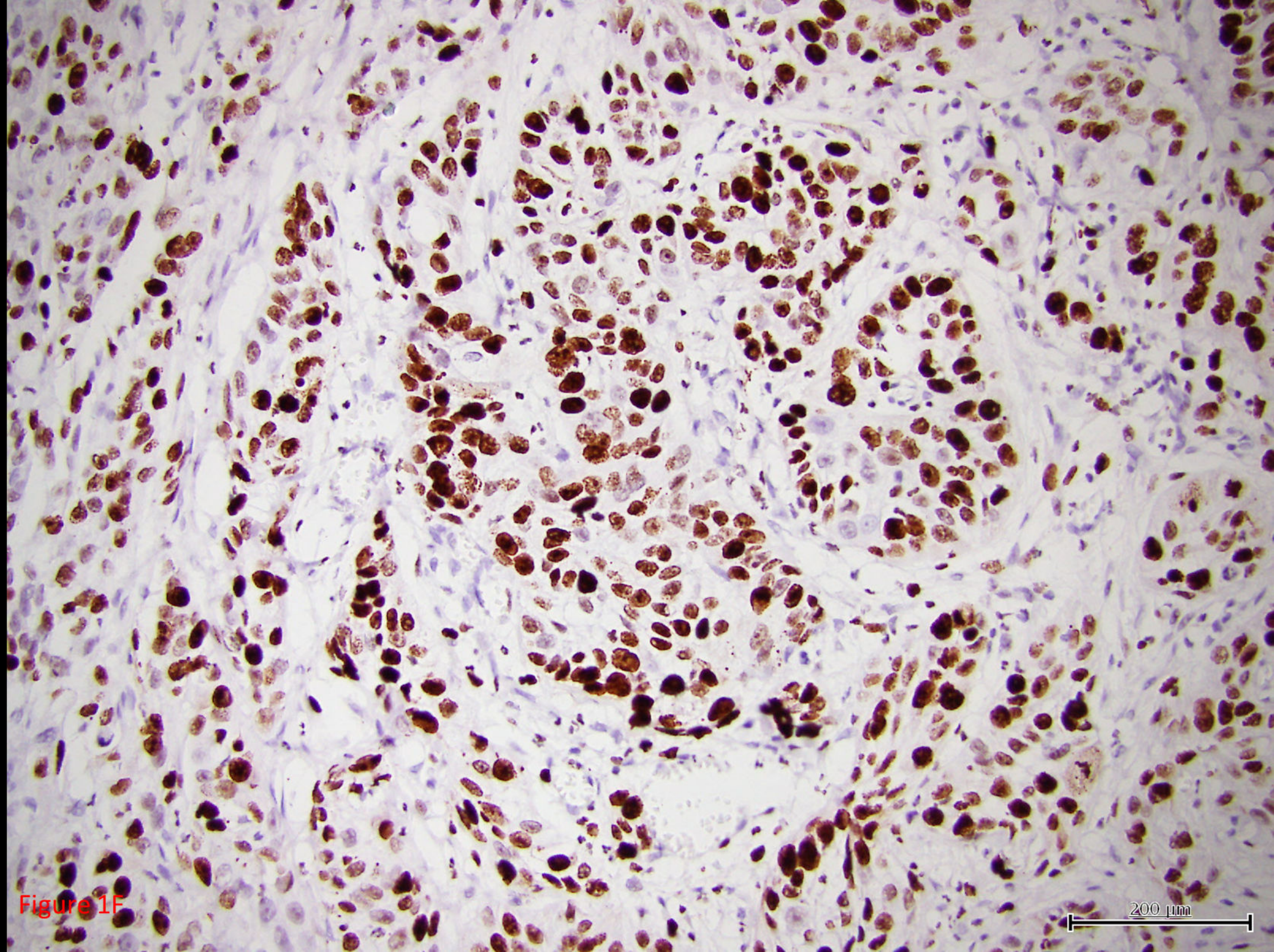

Figure 1F

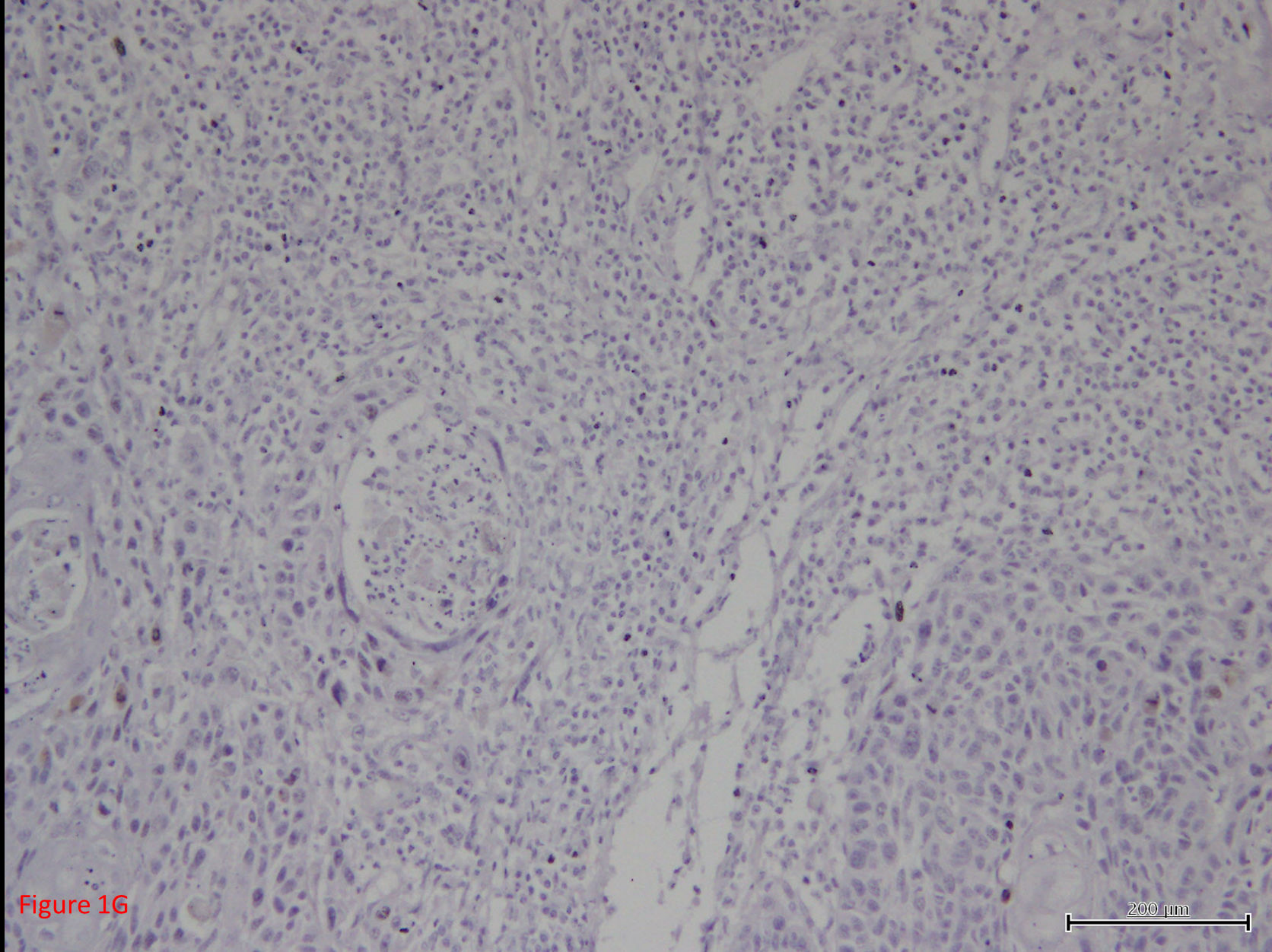

Figure 1G

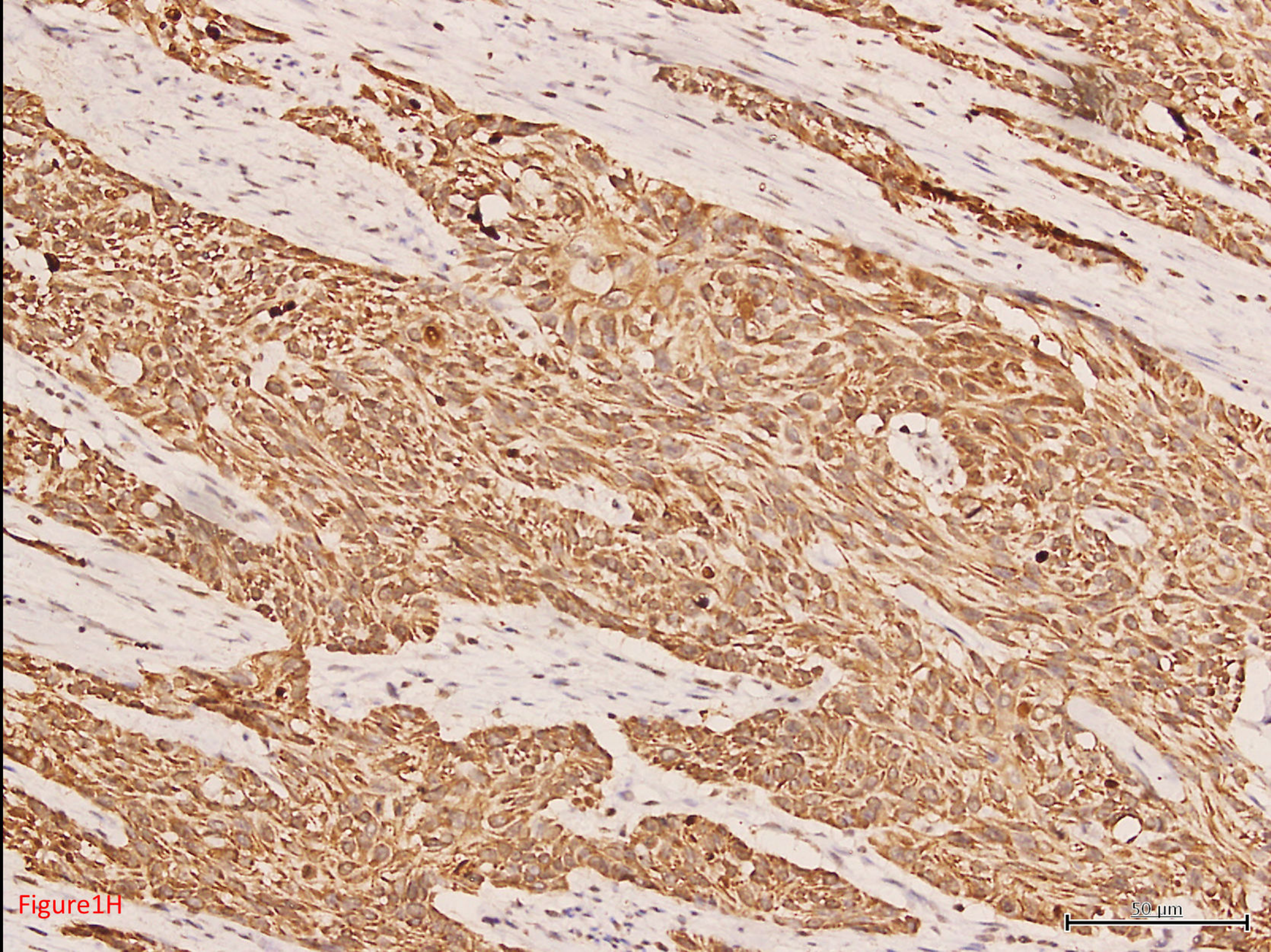

Figure1H

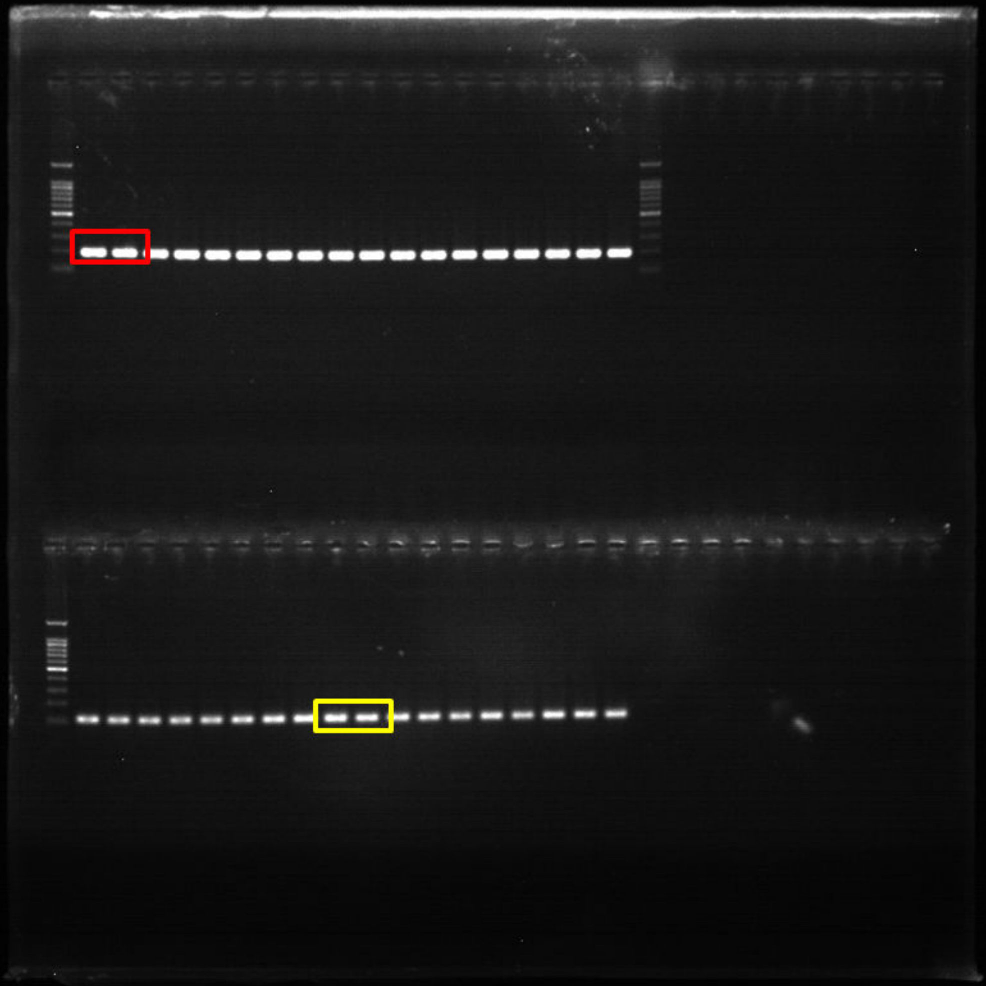

Figure2A(red-GAPDH， yellow-AKR1B10)

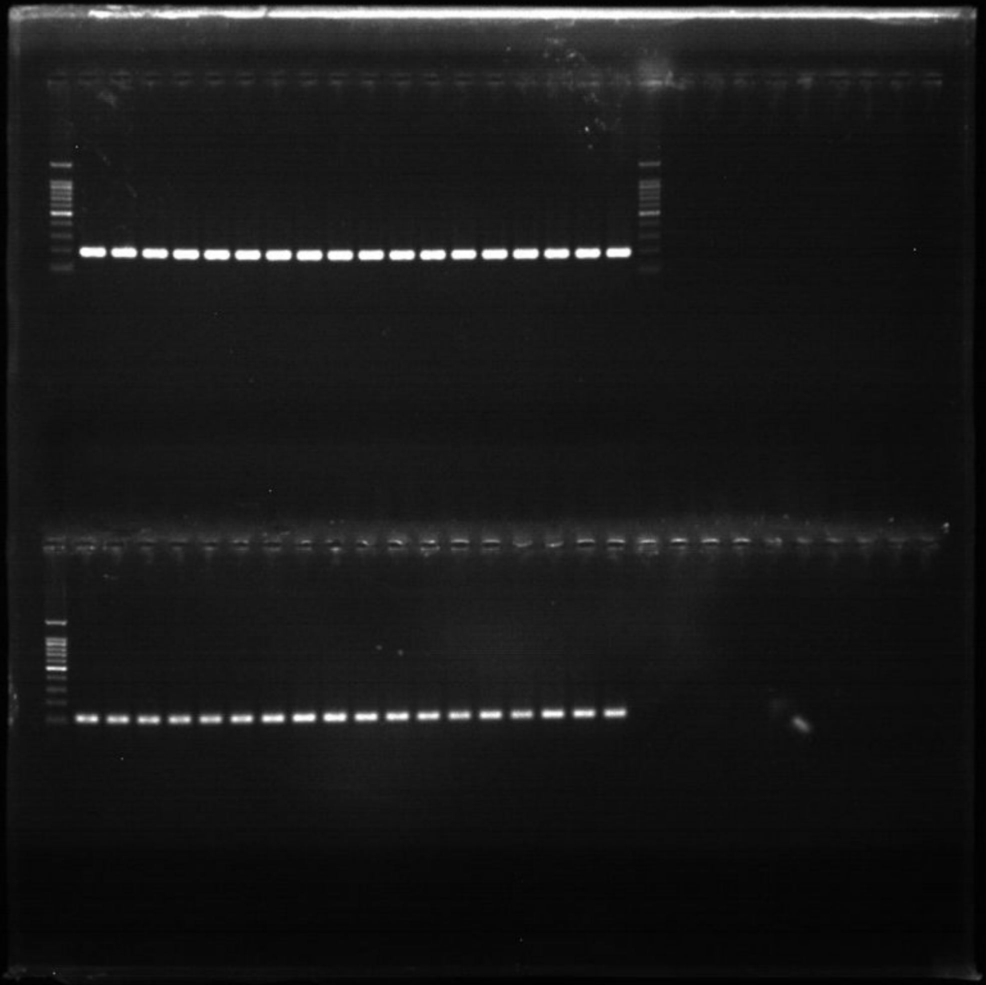

Figure 2A

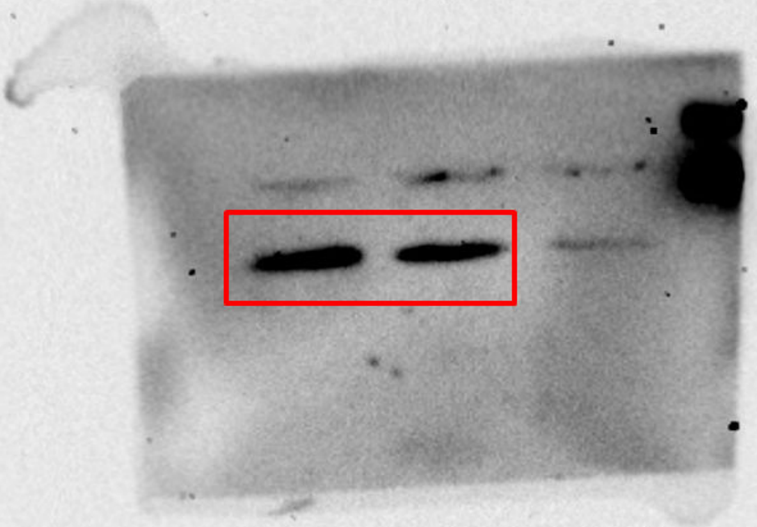

Figure2B(AKR1B10)

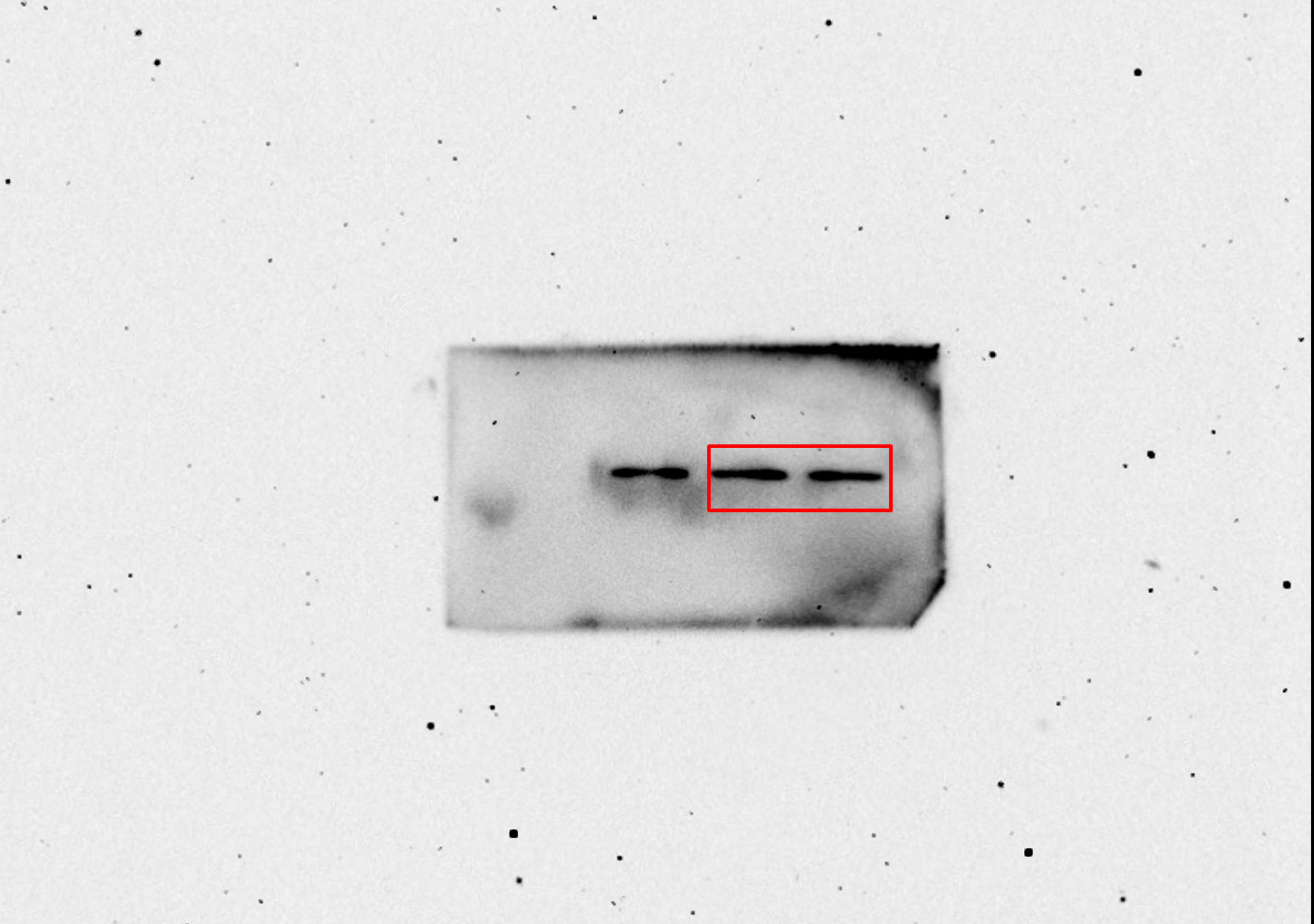

Figure 2B(GAPDH)

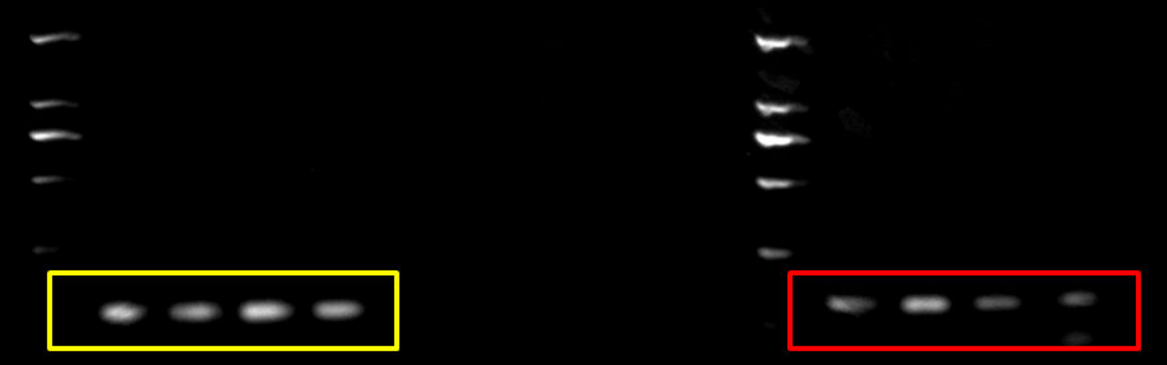

Figure3b(red-AKR1B10,yellow-GAPDH)

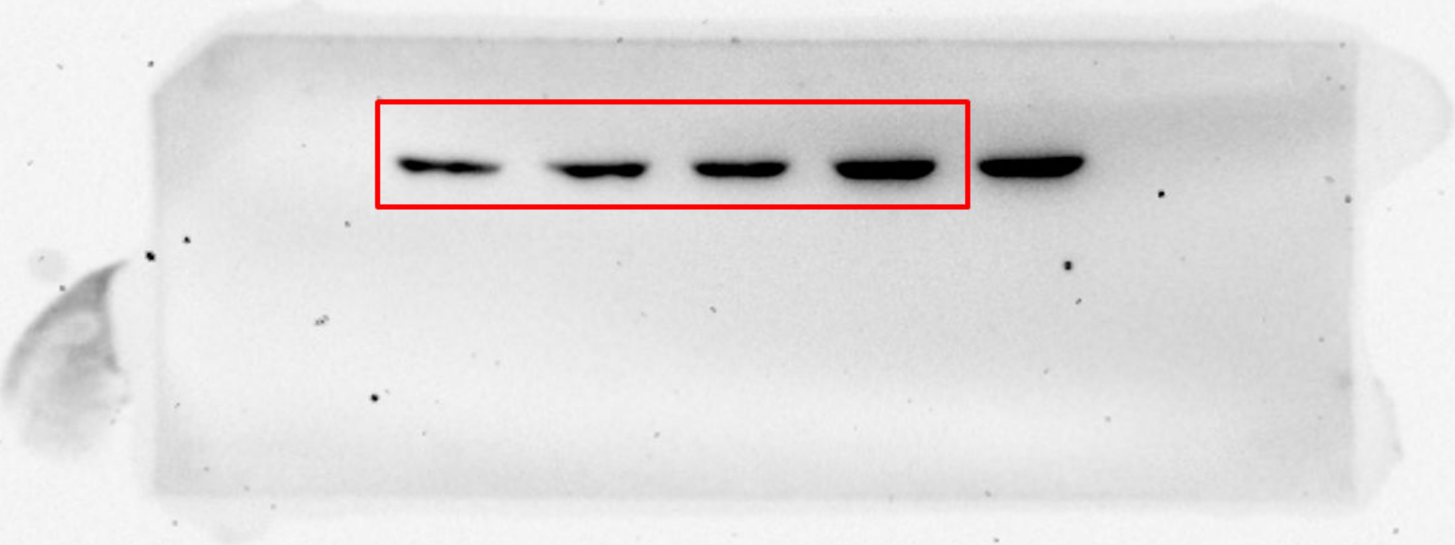

Figure 4A(GAPDH)

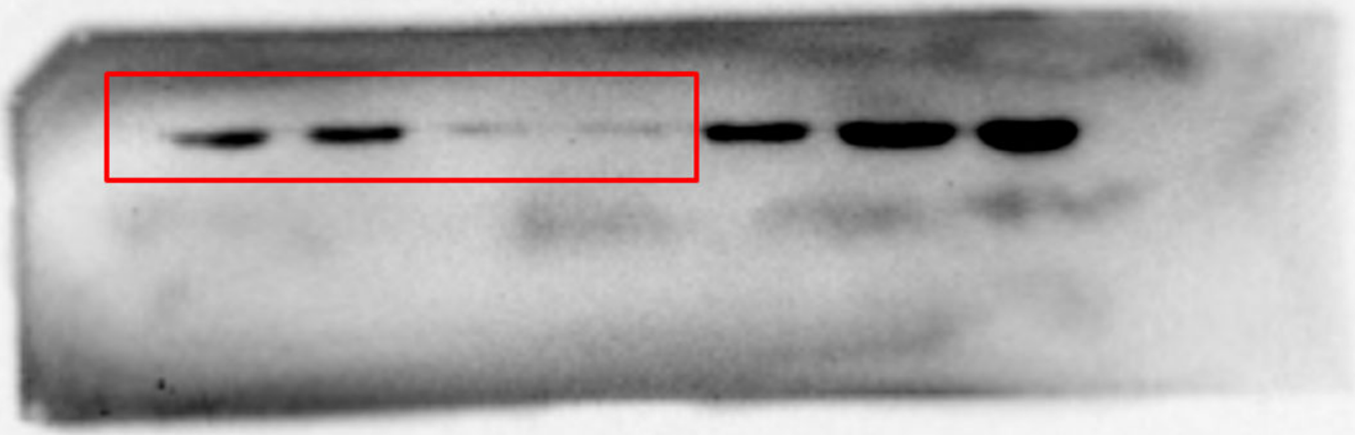

Figure 4A(AKR1B10)

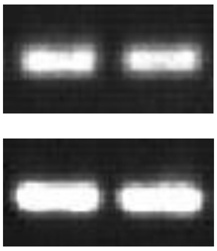

AKR1B10

GAPDH

A549 Hep-2

(Figure2A)

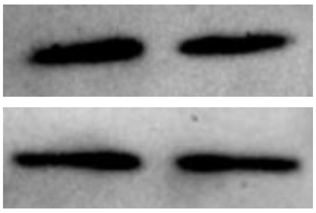

AKR1B10

GAPDH

A549 Hep-2

(Figure2B)

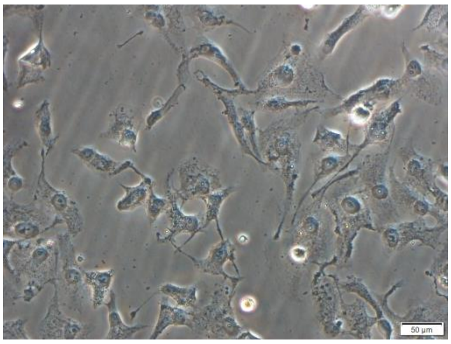

A:Hep-2 (Bright field)

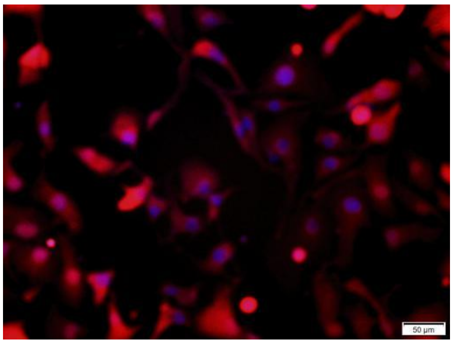

B:Hep-2 (Fluorescence)

(Figure2C)

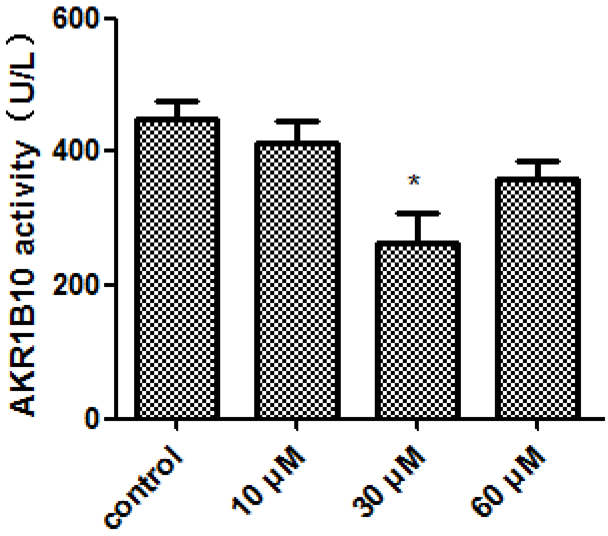

(Figure3A)

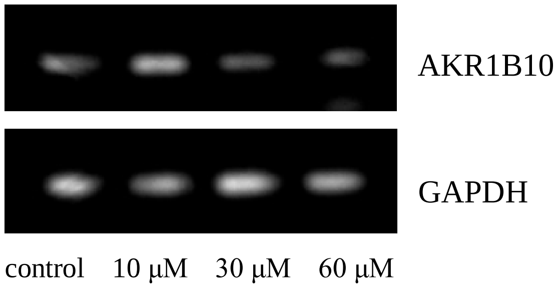

(Figure3B)

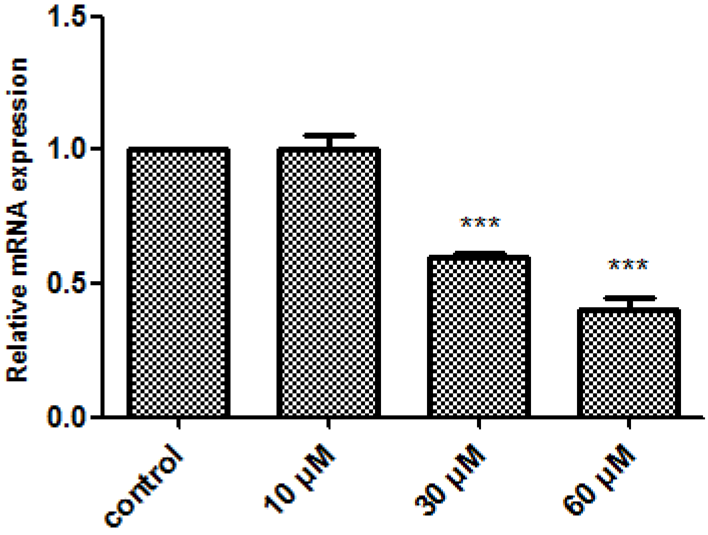

(Figure3C)

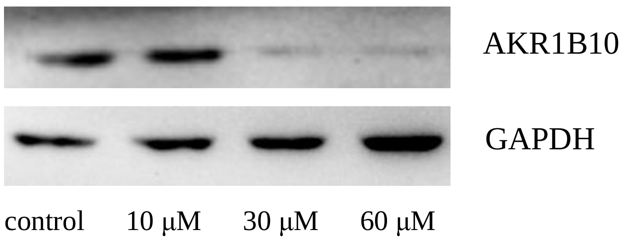

(Figure4A)

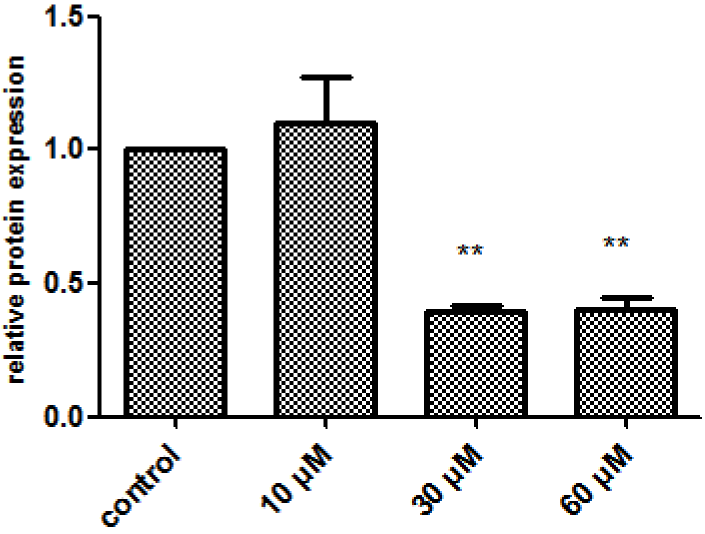

(Figure4B)

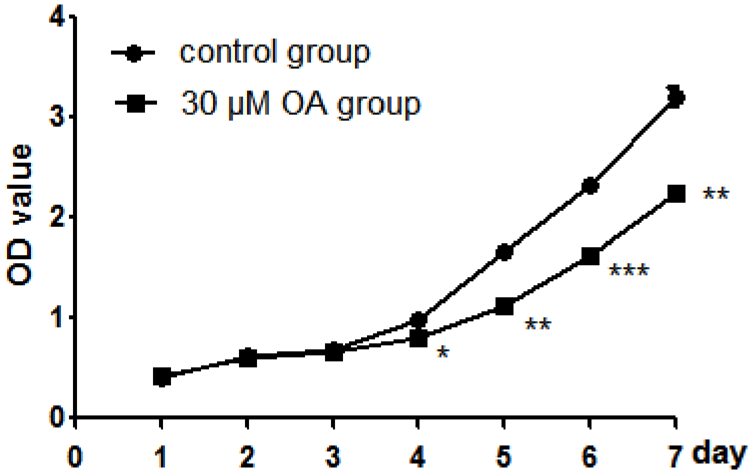

(Figure5A)

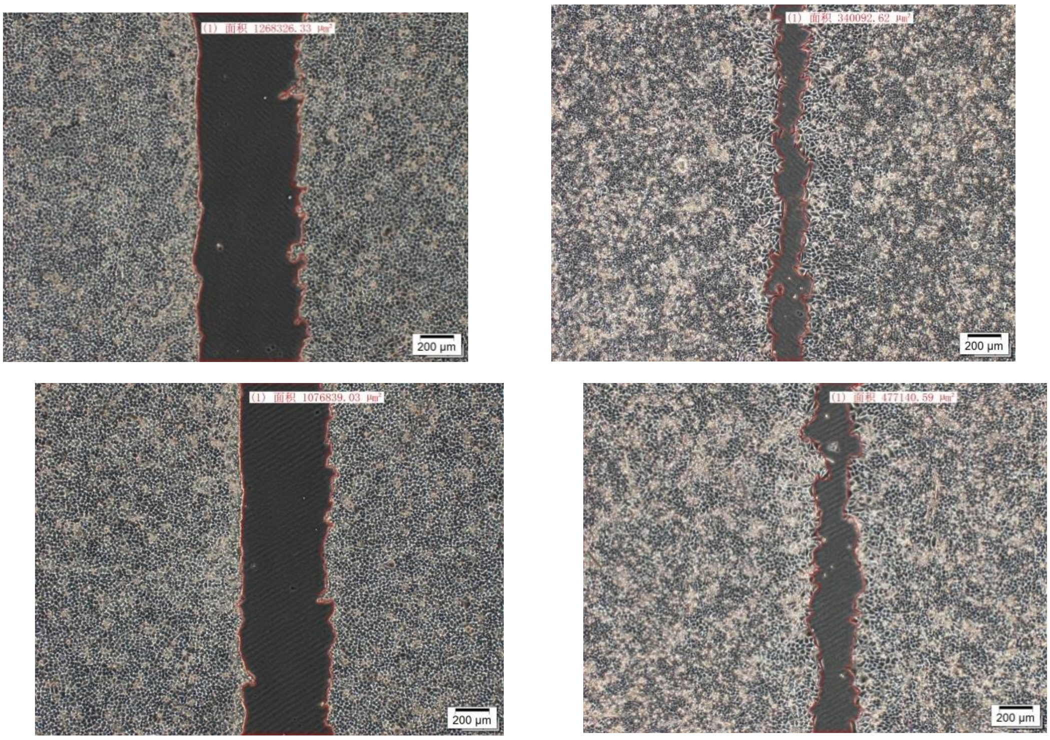
(1) 面积 1268326.33 μm²
200 μm
(1) 面积 340092.62 μm²
200 μm
(1) 面积 1076839.03 μm²
200 μm
(1) 面积 477140.59 μm²
200 μm

(Figure5B)

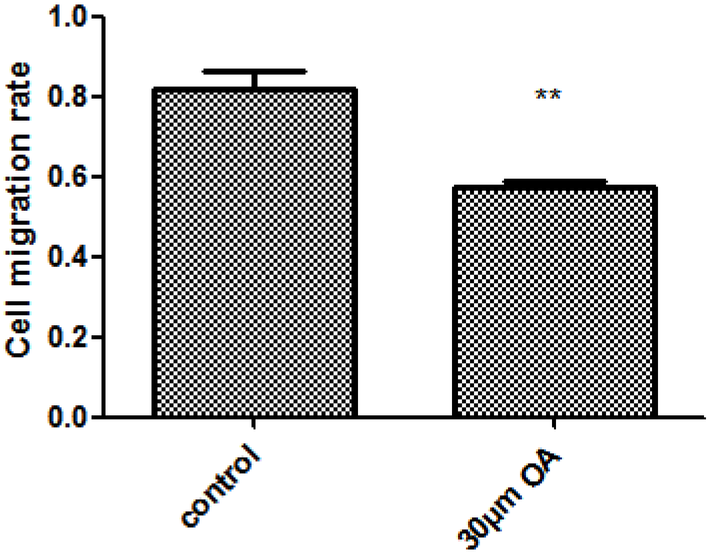
Cell migration rate
1.0
0.8
0.6
0.4
0.2
0.0
**
control
30μm OA

(Figure5C)

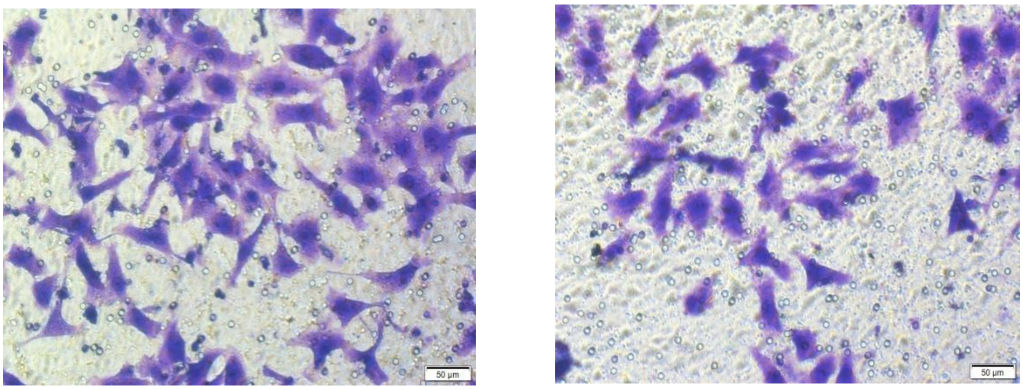

(Figure6A)

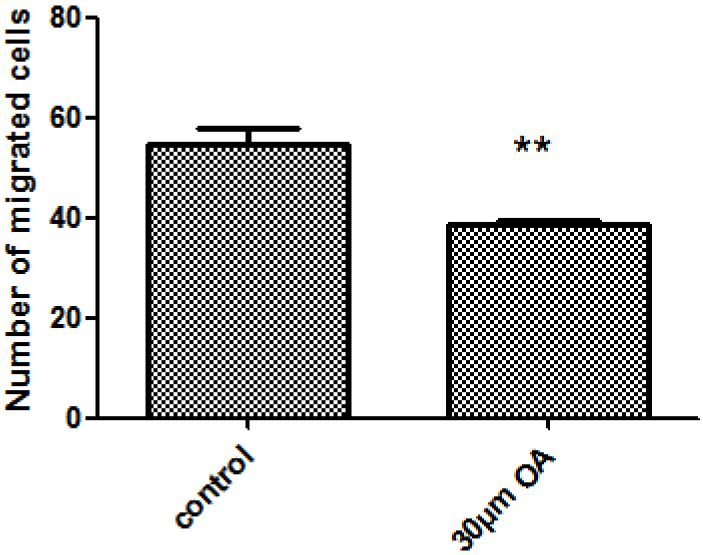

(Figure6B)
